# Supplementary material for: Socioeconomic inequalities in dental caries and their determinants in adolescents in New Delhi, India
Source: BMJ Open. 2014 Dec 12;4(12):e006391. doi: 10.1136/bmjopen-2014-006391 (PMC4267077; doi:10.1136/bmjopen-2014-006391)
Supplement: Author's manuscript [file bmjopen-2014-006391.draft_revisions.pdf]

BMJ Open

Socio-economic inequalities in dental caries and their determinants in adolescents in India

Journal:	*BMJ Open*
Manuscript ID:	bmjopen-2014-006391
Article Type:	Research
Date Submitted by the Author:	18-Aug-2014
Complete List of Authors:	Mathur, Manu; Public Health Foundation of India, Health Promotion<br>Tsakos, Georgios; University College London, Department of Epidemiology and Public Health<br>Millett, Christopher; Imperial College London, Primary Care and Public Health<br>Arora, Monika; Public Health Foundation of India, Health Promotion and Tobacco Control<br>Watt, Richard; University College London, Department of Epidemiology and Public Health
**Primary Subject Heading**:	Dentistry and oral medicine
Secondary Subject Heading:	Dentistry and oral medicine
Keywords:	PUBLIC HEALTH, EPIDEMIOLOGY, SOCIAL MEDICINE

SCHOLARONE™
Manuscripts

Title:

Socio-economic inequalities in dental caries and their determinants in adolescents in India

First and Corresponding Author:

1. **Dr. Manu Raj Mathur**

Public Health Foundation of India

ISID Campus, 4, Institutional Area, Vasant Kunj, New Delhi 110070, India

E Mail: manu.mathur@phfi.org ; Tel: +91-11-49566000

Other Authors:

2. **Dr. Georgios Tsakos**

Department of Epidemiology and Public Health, University College London

1-19 Torrington Place, London, London, WC1E 7HB

E Mail: g.tsakos@phfi.org; Tel: +44-207-6791699

3. **Dr. Christopher Millett**

School of Public Health, Imperial College London

South Kensington Campus, London SW7 2AZ

E Mail: c.millett@imperial.ac.uk ; Tel: +44-207-5895111

For peer review only

4. **Dr. Monika Arora**

Public Health Foundation of India

ISID Campus, 4, Institutional Area, Vasant Kunj, New Delhi 110070, India

E Mail: monika.arora@phfi.org ; Tel: +91-11-49566000

5. **Prof. Richard Watt**

Department of Epidemiology and Public Health, University College London

1-19 Torrington Place, London, London, WC1E 7HB

E Mail: r.watt@phfi.org; Tel: +44-207-6791699

KEYWORDS: socio-economic inequalities, dental caries, adolescents, deprivation, India

WORD COUNT: 3050 words

For peer review only

Title

Socio-economic inequalities in dental caries and their determinants in adolescents in India

For peer review only

ABSTRACT

Objectives: To determine whether there were socioeconomic inequalities in caries experience and decayed teeth of Indian adolescents and assess whether behavioural and psychosocial factors explained these inequalities. **Methods:** Cross sectional study of 1,386 adolescents living in 3 diverse areas of New Delhi. Caries experience and number of decayed teeth were assessed clinically and a questionnaire was used to gather socio-demographic and psychosocial data. Zero Inflated Negative Binomial regression models were used to assess the relationship between the outcomes (caries experience and decayed teeth) and area of residence, adjusting for covariates. **Results:** Significant inequalities in caries experience and number of decayed teeth were observed. Odds of an adolescent being caries free decreased by 66% (OR 0.34, 95% CI 0.23-0.49) and 70% (OR 0.30, 95% CI 0.21-0.43) in adolescents living in resettlement communities or urban slums respectively when compared with the middle class group. No difference was observed among those with caries experience/decayed teeth. Adjusting for covariates did not affect the inequalities. **Conclusions:** Area of residence appears to be a very strong and significant determinant for an adolescent being caries/decay free in India.

For peer review only - http://bmjopen.bmj.com/site/about/guidelines.xhtml

RESEARCH SUMMARY

We studied the presence of socioeconomic inequalities in adolescents and the various factors which influences these inequalities. We found that significant inequalities in caries experience and number of decayed teeth exist among Indian adolescents. Adjusting for behavioural, psychosocial and material factors did not attenuate these inequalities. Relative effect of deprivation on oral health inequalities can be seen only in individuals who are disease free. In order to reduce inequalities in dental caries, there is a need to intervene early and prevent the onset of dental caries.

Strengths

- Very few studies have examined the impact of absolute poverty on the oral health of adolescents in LMICs. This study addresses this gap.
- Fairly large sample size with high response rate.
- Wide array of material, psychosocial and behavioural variables tested through standardized and reliable questionnaire.

Limitations

- This is a cross-sectional study where data was collected at one point of time.
- Risk of reporting and interviewer bias.
- Measurement issues with the scales used for psychosocial variables and for studying material deprivation

For peer review only

INTRODUCTION

Oral diseases are among the most common chronic diseases worldwide[1]. Oral diseases not only have an impact on general health and quality of life but may also increase the risk of mortality[2]. Oral diseases are costly for health systems and individuals, especially in those from low income and deprived households [2].

There are widespread inequalities in oral health outcomes within and between different countries of the world[3]. However, most studies examining social inequalities and gradients in oral health have been conducted in high income countries with populations that generally lie above the poverty line. As such, they could not focus on whether social inequalities exist in context of absolute poverty. No study on oral health inequalities from India has considered populations from extremely deprived areas like urban slums and resettlement communities.

Different theories have highlighted various explanations of inequalities observed in general as well as oral health [4–8]. According to these, inequalities arise because of adverse material circumstances, health-affecting behaviours or due to various psychosocial factors. Although there is a considerable amount of literature in general health[9–11], there is a paucity of evidence in the dental literature examining how different behavioural, psychosocial and socio-environmental factors influence oral health inequalities.

Our study assessed the existence of socioeconomic inequalities in dental caries of adolescents living in different geographical areas and conditions in the city of New Delhi, India. We also explored the effect of material, psychosocial and behavioural determinants on these inequalities in dental caries.

For peer review only

For peer review only - http://bmjopen.bmj.com/site/about/guidelines.xhtml

METHODS

The study was carried out in National Capital Territory (NCT) of Delhi. Nearly 0.2 million people migrate to Delhi every year and the majority of these find habitation in urban slums which constitutes about 20% of the total population of Delhi[12]. Many migrants as well as the urban poor also reside in unauthorized and resettlement communities (settlements which have recently been legalised by the Government and were previously slums and are better off economically in comparison to slums).

Study Population

This cross-sectional study was conducted among adolescents aged 12-15 years living in three diverse residential areas of New Delhi reflecting their economic position: urban slums; resettlement communities; and middle and upper middle class communities.

Study Tools:

Data were collected through an interviewer-administered questionnaire and a clinical examination. The questionnaire measured material resources, neighbourhood social capital, social support, health related behaviours (alcohol and tobacco use, diet, frequency of tooth brushing) and key socio-demographic variables. The questionnaire included pre-existing questions and scales which were checked for reliability and validity in the study population during a pilot study. Material resources were assessed using National Family Health Survey (NFHS), Standard of Living Index which has been validated and used extensively to assess material deprivation in India. Social Capital was measured by a scale developed by Gage et al (2005)[13]. This scale has been adapted from Health Behaviour in School Children (HBSC) study conducted by WHO[14]. Social Support was measured by using Social Support Scale for

For peer review only - http://bmjopen.bmj.com/site/about/guidelines.xhtml

Adolescents (SSAS) developed by Seidman et al (1995)[15]. The questions to assess health-related behaviours in adolescents were derived from the WHO Health Behaviour in School Children (HBSC) survey[14].

A non-invasive clinical examination was performed. We used the DMFT (Diseased Missing & Filled Teeth) Index to measure the level of dental caries and decayed teeth[16]. The DMFT index was calculated on every adolescent using a mouth mirror and a blunt probe. A systematic and standardised approach was used to examine the teeth based upon the WHO criteria [16].

Study Sample:

Slums and resettlement communities were identified from an official list of registered resettlement communities and urban slums. The inclusion criteria were a) communities within a radius of 25 kilometres from the research office, b) both slum and resettlement community present together as a cluster, c) have more than 500 households in each component of the cluster, and d) have a known Non-Governmental Organization working in the community and willing to participate in the research. We identified 14 slums and resettlement communities. A census was performed in each of these communities to collect demographic data.

Adolescents from middle and upper middle class households in India generally study in private schools which have English as medium of education and charge higher fees ('English Medium Schools'). These schools were targeted to obtain the desired sample of adolescents belonging to middle and upper middle class homes. Inclusion criteria for English medium schools were: a) having secondary level classes, b) present in the same vicinity as that of the low income communities in the sampling frame, c) having at least 40 pupils per class, d) being co-educational (both boys and girls).

For peer review only - http://bmjopen.bmj.com/site/about/guidelines.xhtml

We used multi-stage random sampling. Slums and resettlement communities were randomly selected. Amongst the selected communities, all households having at least one child of 12-15 years of age were identified. Eligible households were randomly selected and approached to request adolescents to participate in the study. If a household had 2 or more eligible adolescents then all of them were invited to participate.

English medium private schools were randomly selected from a list of all recognised schools in the study area obtained from Municipal Corporation of Delhi. From the selected schools, grades which normally have children of 12-15 years of age were identified. The class teacher then assigned random numbers to eligible adolescents in order to maintain the anonymity of identity of students. Adolescents were then randomly selected from the provided list of numbers.

Sample size was calculated for difference in means of the two clinical outcomes (caries experience and decayed teeth) between the three different adolescent groups, with 80% power and 5% significance level. The calculated sample size was increased by a factor of 25% to account for potential non-response, and then by a factor of 1.3 to account for the effect of clustering. The estimated sample size was 1338 adolescents (446 adolescents per group).

Variables

Main explanatory variable in this study was adolescent socioeconomic position assessed through area of residence (slums, resettlement community, middle and upper middle class homes). Covariates are grouped into the following categories; a) material resources, b) neighbourhood social capital (bridging and bonding types of social capital measuring trust, norms and reciprocity in a community), c) social support and d) health related behaviours (diet, tobacco &

For peer review only - http://bmjopen.bmj.com/site/about/guidelines.xhtml

alcohol use, brushing frequency, visit to a dentist, getting bullied & involvement in physical fight). Outcome variables were dental caries experience and prevalence of decayed teeth.

Data Analysis

Descriptive statistics were calculated to assess the frequency distributions of explanatory and outcome variables. As there were a high number of zeros (caries/ decay free teeth) in the outcomes and the variance was considerably greater than the mean, Zero Inflated Negative Binomial (ZINB) regression analysis was used to assess the association between area of residence and caries experience (DMFT) and number of carious teeth (DT) [17]. A ZINB regression generates two separate models. The first model is a logit model generated for a ''certain zero'' in the outcome (i.e. no decayed, missing or filled teeth if DMFT, or no active caries if DT is the outcome), predicting whether or not an adolescent would be in this group. The second model is the negative binomial model predicting the count of those adolescents who are not in "certain zero group", that is looking at the number of decayed, missing or filled teeth among those with DMFT>0 or at the number of decayed teeth among those with DT>0 respectively.

Ethical approval was granted from University College London (UCL) Research Ethics Committee and the Public Health Foundation of India (PHFI) Technical Review and Institutional Ethics Committee.

For peer review only - http://bmjopen.bmj.com/site/about/guidelines.xhtml

RESULTS

A response rate of 86.6% was achieved (n = 1386). There were 736 (53.1%) boys and 650 (46.9%) girls; proportions that are almost the same to the gender distribution of Delhi NCT (53.6% males and 46.4% females) [18]. Overall, 460 (33.2%) adolescents belonged to middle and upper middle class group, 462 (33.3%) were from resettlement communities and 464 (33.5%) from urban slums. Almost half (49.7%) of the clinically examined adolescents had previous caries experience. The mean DMFT was 1.36 (1.27, 1.46). Out of the 689 adolescents with caries experience, 644 had decayed teeth at the time of clinical examination (mean = 1.21; 95%CI: 1.12, 1.31).

There was a clear social gradient, with consistently greater levels of caries experience (DMFT) at each lower level of area of residence of adolescents ($p < 0.0001$). Adolescents from middle/ upper middle class homes had mean DMFT of 0.96 (95%CI: 0.82, 1.21), those from resettlement communities had a mean of 1.38 (95% CI 1.23-1.54) and those from urban slums had a mean DMFT of 1.74 (95%CI: 1.55, 1.93). Similarly, the mean number of decayed teeth was higher at each lower socioeconomic group ($p < 0.0001$). The mean number of decayed teeth in adolescents from middle/ upper middle class homes was 0.72 (0.59, 0.85), in those from resettlement communities 1.34 (1.19, 1.50) and in those from urban slums 1.58 (1.40, 1.76) (Table 1, Figure 1).

For peer review only - http://bmjopen.bmj.com/site/about/guidelines.xhtml

Table - 1

Socioeconomic Inequalities in Caries Experience & Decayed Teeth

**Variable**	DMFT		Carious Teeth	
	Mean (95% CI)	p-value	Mean (95% CI)	p-value
**Middle/ Upper Middle Class**	0.96 (0.82-1.21)	<.0001	0.72 (0.59-0.85)	<.0001
**Resettlement Communities**	1.38 (1.23-1.54)		1.34 (1.19-1.50)	
**Slums**	1.74 (1.55-1.93)		1.58 (1.40-1.76)	

Table 2 shows the results of the ZINB regression models for caries experience. Adjustment for covariates did not have a considerable effect on the inequalities identified. Compared to the middle/upper middle class adolescents, those living in resettlement communities had a significantly lower odds of being caries-free (OR: 0.33; 95%CI: 0.23, 0.49 in the unadjusted model, OR: 0.22; 95%CI: 0.12, 0.39 in fully adjusted model), and the same was the case for those living in urban slums (OR: 0.30, 95%CI: 0.21, 0.43 in unadjusted and OR: 0.22, 95%CI: 0.11, 0.46 in fully adjusted models). In contrast, there were no differences between the three residential sites in relation to the number of teeth with caries experience (DMFT > 0).

For peer review only

Table 2

Association between area of residence and caries experience adjusting for demographic variables, health related behaviours, material resources, social support and social capital (N = 1386)

	Area of Residence		
	**Middle Class**	**Resettlement Community**	**Slums**
		**O.R (95% CI)**	
Logit			
**Unadjusted**	1	0.34 (0.23-0.49)***	0.30 (0.21-0.43)***
**Adjusted for Age, Sex and Religion**	1	0.28 (0.19-0.41)***	0.25 (0.17-0.37)***
**Fully Adjusted for all covariates##**	1	0.22(0.12-0.39)***	0.22 (0.11-0.46)***
		**I.R.R (95% CI)**	
Negative Binomial			
**Unadjusted**	1	0.88 (0.73-1.05)	1.06 (0.89-1.26)
**Adjusted for Age, Sex and Religion**	1	0.91 (0.76-1.09)	1.13 (0.94-1.35)
**Fully Adjusted for all covariates##**	1	1.00 (0.76-1.34)	1.10(0.78-1.54)

# Association tested using Zero Inflated Negative Binomial Regression Method

## Fully adjusted for age, sex, religion, health related behaviours, material resources, social support and social capital

***** p< 0.001**

The respective results for decayed teeth were similar, with a significantly lower odds of being decay-free for adolescents from resettlement communities (OR: 0.25; 95% CI: 0.17-0.37) and those from urban slums (OR: 0.24; 95% CI: 0.16-0.35) compared to their more affluent counterparts. The fully adjusted odds ratios were 0.21 ((5% CI: 0.14, 0.32) for adolescents from resettlement communities and urban slums in comparison to adolescents from middle/upper

For peer review only - http://bmjopen.bmj.com/site/about/guidelines.xhtml

middle class homes. There were no significant differences between the three groups in the number of decayed teeth among those with current decay (Table 3).

Table 3

Association between area of residence and decayed teeth adjusting for demographic variables, health related behaviours, material resources, social support and social capital (N = 1386)

	Area of Residence		
	**Middle Class**	**Resettlement Community**	**Slums**
		**O.R (95% CI)**	
Logit			
**Unadjusted**	1	0.25 (0.17-0.37)***	0.24 (0.16-0.35)***
**Adjusted for Age, Sex and Religion**	1	0.21 (0.14-0.31)***	0.20 (0.13-0.30)***
**Fully Adjusted for all covariates**$^{\#\#}$	1	0.21(0.14-0.32)***	0.21 (0.14-0.32)***
		**I.R.R (95% CI)**	
Negative Binomial			
**Unadjusted**	1	0.95 (0.78-1.17)	1.11(0.91-1.35)
**Adjusted for Age, Sex and Religion**	1	1.02 (0.83-1.25)	1.21 (0.99-1.47)
**Fully Adjusted for all covariates**$^{\#\#}$	1	1.01 (0.82-1.24)	1.19(0.97-1.46)

# Association tested using Zero Inflated Negative Binomial Regression Method

## Fully adjusted for age, sex, religion, health related behaviours, material resources, social support and social capital

***** p< 0.001**

For peer review only - http://bmjopen.bmj.com/site/about/guidelines.xhtml

DISCUSSION

We observed a monotonic gradient for the differences in caries experience and decayed teeth between adolescents living in diverse residential areas of New Delhi. Our results showed that there is a significant difference between the proportion of individuals who were caries free or individuals with decay free teeth between the three areas of residence. However, once an individual experienced caries or developed tooth decay, it did not matter as to which residential group they belonged to as there were no significant differences in the probability of having one or more carious or decayed teeth between the three groups.

The study population comprised of adolescents from areas with extreme deprivation, such as urban slums. Only two studies have looked at the socioeconomic inequalities in dental caries in India [19,20], but they did not study adolescents from extremely deprived areas. Our findings in relation to socioeconomic inequalities observed in caries experience and number of decayed teeth are similar to previous studies conducted on adolescents[21–25], and children [26–29].

We used area of residence as an indicator of socioeconomic position of adolescents. Only one previous study has looked at inequalities in dental caries using an area based measure of socioeconomic position[27]. Thomson and Mackay (2004) in their study on 9-year-old school children from New Zealand, used both area based and individual based measures of socioeconomic position and found that the inequalities in dental caries were steeper when area based measures were used to define socioeconomic position.

Adjusting for all others factors simultaneously in our study did not caused a noteworthy change in the associations, showing that the combination of different factors investigated in this study had a limited effect on the observed inequalities in dental caries. Jung et al (2011) showed that

For peer review only

behavioural (brushing frequency, diet, smoking and alcohol use) and family affluence had no influence on the socioeconomic inequalities observed for self-reported toothache, bad breath and fractured teeth in South Korean adolescents. However, when combined with psychosocial factors (perceived stress and happiness), these factors partially accounted for the inequalities in oral health[30].

We collected data on both oral hygiene related (tooth brushing frequency, dental visit frequency) and health affecting (tobacco, alcohol, involvement in physical fight, diet) behaviours to understand the effect of behaviours on socioeconomic inequalities in oral health. None of the health affecting behaviours had any significant effect on inequalities in oral health observed in our study. Most of the studies on adolescents and young children have also shown a negligible or a minor effect of health related behaviours on inequalities in oral health[24,31,32].

Material deprivation in our study was measured through the NFHS standard of living index [33]. This index was first developed in 2000 and assessed the availability of basic material things required for living by an individual. However, India has since seen rapid economic development leading to a general improvement in the standard of living. Therefore, some of the items and respective weights used in the standard of living index may not be equally relevant in the current situation. This measurement issue may partly explain why we were not able to see any effect of material deprivation on inequalities in dental caries.

Social capital is a multidimensional concept described by different authors in different ways, therefore not easily measured with only a few items [34]. Putnam (2000) in his description of social capital stressed that community participation is also an intertwined feature along with trust and norms of reciprocity and forms an important component of social capital. The social capital questionnaire[13] used in our study measured the trust and norms of reciprocity in the society but

For peer review only - http://bmjopen.bmj.com/site/about/guidelines.xhtml

did not measure the level of community participation which might be one of the reasons of not finding any effect of social capital on inequalities in caries experience and decayed teeth. While social support may be seen as bi-directional (receiving as well as giving)[36], our scale of social support[15] measured mainly the received support or support available to an adolescent and did not assess the aspect of 'giving' support to others.

Adolescents were sampled from extremely deprived urban slums and deprived resettlement areas of New Delhi thus providing a realistic reflection of oral health inequalities in urban areas. We adopted scales and questions from internationally validated questionnaires, and further tested and adapted them for use on Indian adolescent populations. We acknowledge a number of study limitations in addition to the measurement issues about material deprivation and psychosocial variables described above. We studied only social capital and social support from the vast array of psychosocial variables. There are many other psychosocial variables like stress, depression and anxiety which were not investigated. Thus the results do not fully cover the entire psychosocial pathway to oral health inequalities. Furthermore, reporting bias cannot be ruled out as adolescents who were well versed with the consent form and objectives of the study might have given responses that are either socially desirable or perceived to be "wanted" by the interviewer.

Our study indicates towards a very important health promotion message. It highlights the fact that the relative effect of deprivation on oral health inequalities can be seen only in individuals who are disease free. Once the disease sets in, there are no differences between adolescents based on their socio-economic position. Our study has also shown that area of residence may be a very important determinant of the oral health status of adolescents in India. Psychosocial, material or behavioural characteristics did not mediate the role of extreme living conditions on

For peer review only - http://bmjopen.bmj.com/site/about/guidelines.xhtml

oral health. This finding highlights the importance of a primary prevention approach[37], in reducing inequalities in oral health. In order to reduce inequalities in dental caries, there is a need to intervene early and prevent the onset of dental caries and 'act before it happens' rather than intervening after caries has affected the population. There is a need to design policies which aim at primary prevention and improving health by taking action on the broader structural determinants of health.

AUTHOR'S CONTRIBUTIONS

MRM, RGW, GT and MA conceptualized and designed the study. MRM collected the data, performed analysis and wrote the manuscript. RGW and GT directed the development of methodology, analytic plan and interpretation of results. MRM, RW, GT and CM undertook the critical revisions of the paper for substantial intellectual content. MA contributed to the background and methods section of this paper. All the authors approved of the final version to be published.

COMPETING INTEREST

We declare no competing interest.

ACKNOWLEDGEMENTS/ FUNDING

This work was supported by a Wellcome Trust Capacity Strengthening Strategic Award to the Public Health Foundation of India and a consortium of UK universities.

DATA SHARING

No additional data available

For peer review only

For peer review only - http://bmjopen.bmj.com/site/about/guidelines.xhtml

REFERENCES:

1 Petersen PE. Priorities for research for oral health in the 21st century--the approach of the WHO Global Oral Health Programme. *Community dental health* 2005;**22**:71–4.

2 Petersen PE. The World Oral Health Report 2003 WHO Global Oral Health Programme. *Oral Health* 2003;**31 Suppl 1**:3–23.

3 Marmot M. Social determinants of health inequalities. *Lancet* 2005;**365**:1099–104. doi:10.1016/S0140-6736(05)71146-6

4 Watt RG. Emerging theories into the social determinants of health: implications for oral health promotion. *Community dentistry and oral epidemiology* 2002;**30**:241–7.

5 Sisson KL. Theoretical explanations for social inequalities in oral health. *Community dentistry and oral epidemiology* 2007;**35**:81–8. doi:10.1111/j.1600-0528.2007.00354.x

6 Black D., Townsend P, Davidson N. *Inequalities in health : the Black report*. Harmondsworth: : Penguin 1980.

7 Whitehead M. *Inequalities in Health: The Black Report and the Health Divide*. Harmondsworth: : Penguin 1986.

8 Bartley M. *Health Inequality: an introduction to theories, concepts and methods*. Oxford: : Blackwell Publishing Limited 2004.

9 Karriker-Jaffe KJ, Foshee VA, Ennett ST, *et al.* Sex differences in the effects of neighborhood socioeconomic disadvantage and social organization on rural adolescents' aggression trajectories. *American journal of community psychology* 2009;**43**:189–203. doi:10.1007/s10464-009-9236-x

10 Kohen DE, Leventhal T, Dahinten VS, *et al.* Neighborhood disadvantage: pathways of effects for young children. *Child development* 2008;**79**:156–69. doi:10.1111/j.1467-8624.2007.01117.x

11 Sabbah W, Watt RG, Sheiham A, *et al.* The role of cognitive ability in socio-economic inequalities in oral health. *Journal of Dental Research* 2009;**88**:351–5.

12 MoHFW. STATE OF URBAN HEALTH IN DELHI. New Delhi: 2007.

13 Gage JC, Overpeck MD, Nansel TR, *et al.* Peer activity in the evenings and participation in aggressive and problem behaviors. *The Journal of adolescent health : official publication of the Society for Adolescent Medicine* 2005;**37**:517. doi:10.1016/j.jadohealth.2004.12.012

For peer review only - http://bmjopen.bmj.com/site/about/guidelines.xhtml

14 Roberts C, Freeman J, Samdal O, *et al.* The Health Behaviour in School-aged Children (HBSC) study: methodological developments and current tensions. *International journal of public health* 2009;**54 Suppl 2**:140–50. doi:10.1007/s00038-009-5405-9

15 Seidman E, Allen L, Aber JL, *et al.* Development and validation of adolescent-perceived microsystem scales: social support, daily hassles, and involvement. *American journal of community psychology* 1995;**23**:355–88.

16 WHO. *Oral Health Surveys; Basic Methods*. 4th Editio. Geneva: : World Health Organization 1997.

17 Lewsey JD, Thomson WM. The utility of the zero-inflated Poisson and zero-inflated negative binomial models: a case study of cross-sectional and longitudinal DMF data examining the effect of socio-economic status. *Community dentistry and oral epidemiology* 2004;**32**:183–9. doi:10.1111/j.1600-0528.2004.00155.x

18 Census of India. Tables and Statements. 2011.http://www.censusindia.gov.in/2011-prov-results/data_files/delhi/3_PDFC-Paper-1-tables_60_81.pdf (accessed 3 Nov2012).

19 Christian B, Evans RW. Has urbanization become a risk factor for dental caries in Kerala, India: a cross-sectional study of children aged 6 and 12 years. *International journal of paediatric dentistry* 2009;**19**:330–7. doi:10.1111/j.1365-263X.2009.00979.x

20 Singh A, Sequiera P, Acharya S, *et al.* Oral health status of two 12-year-old socially disadvantaged groups in South India: a comparative study. *Oral health & preventive dentistry* 2011;**9**:3–7.

21 Armfield JM. Socioeconomic inequalities in child oral health: a comparison of discrete and composite area-based measures. *Journal of public health dentistry* 2007;**67**:119–25.

22 Freire MC, Sheiham A, Hardy R. Adolescents' sense of coherence, oral health status, and oral health-related behaviours. *Community Dentistry & Oral Epidemiology* 2001;**29**:204–12.

23 Perera I, Ekanayake L. Factors influencing perception of oral health among adolescents in Sri Lanka. *International Dental Journal* 2008;**58**:349–55.

24 Polk DE, Weyant RJ, Manz MC. Socioeconomic factors in adolescents' oral health: are they mediated by oral hygiene behaviors or preventive interventions? *Community dentistry and oral epidemiology* 2010;**38**:1–9. doi:10.1111/j.1600-0528.2009.00499.x

25 Zurriaga O, Martíínez-Beneito MA, Abellán JJ, *et al.* Assessing the social class of children from parental information to study possible social inequalities in health outcomes. *Annals of epidemiology* 2004;**14**:378–84. doi:10.1016/j.annepidem.2003.06.001

For peer review only

For peer review only - http://bmjopen.bmj.com/site/about/guidelines.xhtml

26 Poulton R, Caspi A, Milne BJ, *et al.* Association between children's experience of socioeconomic disadvantage and adult health: a life-course study. *Lancet* 2002;**360**:1640–5. doi:10.1016/S0140-6736(02)11602-3

27 Thomson WM, Mackay TD. Child dental caries patterns described using a combination of area-based and household-based socio-economic status measures. *Community Dental Health* 2004;**21**:285–90.

28 Sagheri D, McLoughlin J, Clarkson JJ. The prevalence of dental caries and fissure sealants in 12 year old children by disadvantaged status in Dublin (Ireland). *Community Dental Health* 2009;**26**:32–7.

29 Van den Branden S, Van den Broucke S, Leroy R, *et al.* Effects of time and socio-economic status on the determinants of oral health-related behaviours of parents of preschool children. *European Journal of Oral Sciences* 2012;**120**:153–60.

30 Jung SH, Watt RG, Sheiham A, *et al.* Exploring pathways for socioeconomic inequalities in self-reported oral symptoms among Korean adolescents. *Community Dentistry and Oral Epidemiology* 2011;**39**:221–9.

31 Ravaghi V, Underwood M, Marinho V, *et al.* Socioeconomic status and self-reported oral health in Iranian adolescents: The role of selected oral health behaviors and psychological factors. *Journal of Public Health Dentistry* Published Online First: 2012.

32 Sanders AE, Spencer a J, Slade GD. Evaluating the role of dental behaviour in oral health inequalities. *Community dentistry and oral epidemiology* 2006;**34**:71–9. doi:10.1111/j.1600-0528.2006.00261.x

33 Vyas S, Kumaranayake L. Constructing socio-economic status indices: how to use principal components analysis. *Health policy and planning* 2006;**21**:459–68. doi:10.1093/heapol/czl029

34 Berry HL, Welsh JA. Social capital and health in Australia: An overview from the household, income and labour dynamics in Australia survey. *Social science & medicine (1982)* 2010;**70**:588–96. doi:10.1016/j.socscimed.2009.10.012

35 Putnam R. *Bowling Alone: The Collapse and Revival of American Community*. New York: : Simon and Schuster 2000.

36 Kahn RL, Antonucci TC. Convoys over the lifecourse; attachment roles and social support. In: Baltes PB, Brim O, eds. *Life span development and behaviour*. New York: : Academic Press 1980. 253–86.

37 WHO. The Ottawa Charter for Health Promotion. Geneva: 1986.

For peer review only - http://bmjopen.bmj.com/site/about/guidelines.xhtml

Figure

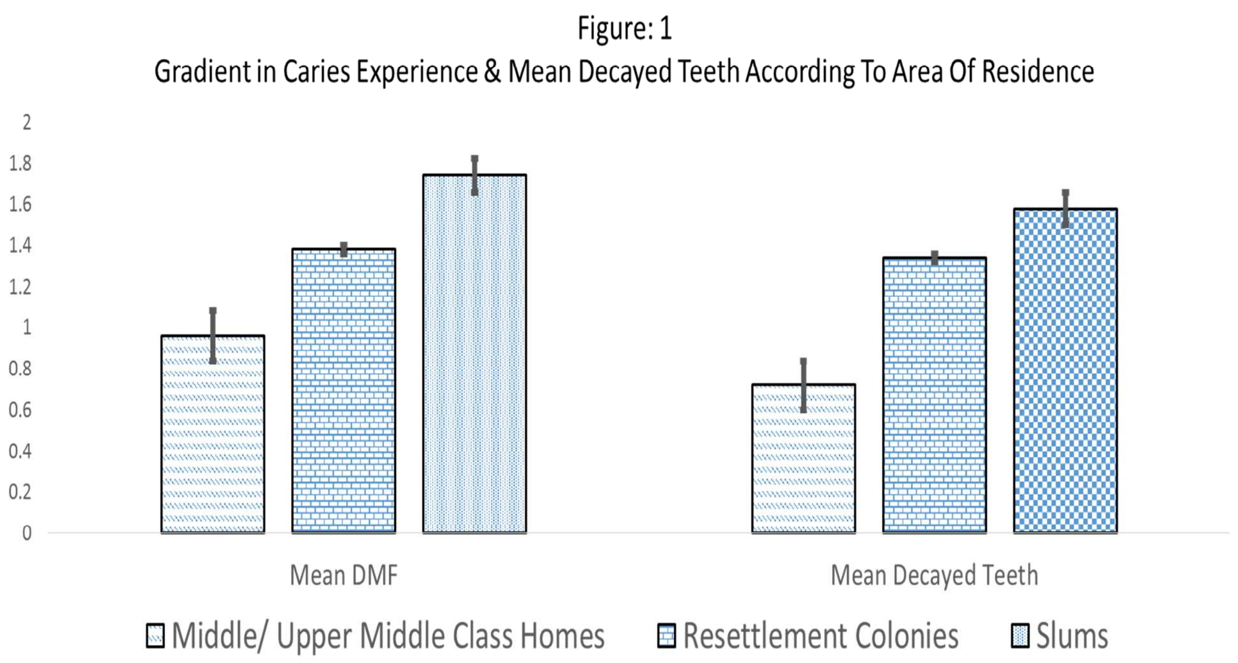
Figure: 1
Gradient in Caries Experience & Mean Decayed Teeth According To Area Of Residence
2
1.8
1.6
1.4
1.2
1
0.8
0.6
0.4
0.2
0
Mean DMF
Mean Decayed Teeth
Middle/ Upper Middle Class Homes
Resettlement Colonies
Slums

STROBE Statement—checklist of items that should be included in reports of observational studies

	Item No	Recommendation	Included in Text
**Title and abstract**	1	(*a*) Indicate the study's design with a commonly used term in the title or the abstract	Yes
		(*b*) Provide in the abstract an informative and balanced summary of what was done and what was found	Yes
**Introduction**			
Background/rationale	2	Explain the scientific background and rationale for the investigation being reported	Yes
Objectives	3	State specific objectives, including any prespecified hypotheses	Yes
**Methods**			
Study design	4	Present key elements of study design early in the paper	Yes
Setting	5	Describe the setting, locations, and relevant dates, including periods of recruitment, exposure, follow-up, and data collection	Yes
Participants	6	(*a*) *Cohort study*—Give the eligibility criteria, and the sources and methods of selection of participants. Describe methods of follow-up *Case-control study*—Give the eligibility criteria, and the sources and methods of case ascertainment and control selection. Give the rationale for the choice of cases and controls *Cross-sectional study*—Give the eligibility criteria, and the sources and methods of selection of participants	Yes
		(*b*) *Cohort study*—For matched studies, give matching criteria and number of exposed and unexposed *Case-control study*—For matched studies, give matching criteria and the number of controls per case	NA
Variables	7	Clearly define all outcomes, exposures, predictors, potential confounders, and effect modifiers. Give diagnostic criteria, if applicable	Yes
Data sources/ measurement	8*	For each variable of interest, give sources of data and details of methods of assessment (measurement). Describe comparability of assessment methods if there is more than one group	*Yes*
Bias	9	Describe any efforts to address potential sources of bias	Yes
Study size	10	Explain how the study size was arrived at	Yes

For peer review only

Quantitative variables	11	Explain how quantitative variables were handled in the analyses. If applicable, describe which groupings were chosen and why	Yes
Statistical methods	12	(*a*) Describe all statistical methods, including those used to control for confounding	Yes
		(*b*) Describe any methods used to examine subgroups and interactions	NA
		(*c*) Explain how missing data were addressed	NA
		(*d*) *Cohort study*—If applicable, explain how loss to follow-up was addressed<br>*Case-control study*—If applicable, explain how matching of cases and controls was addressed<br>*Cross-sectional study*—If applicable, describe analytical methods taking account of sampling strategy	Yes
		(*e*) Describe any sensitivity analyses	

Continued on next page

For peer review only

**Results**			
Participants	13*	(a) Report numbers of individuals at each stage of study—eg numbers potentially eligible, examined for eligibility, confirmed eligible, included in the study, completing follow-up, and analysed	Yes
		(b) Give reasons for non-participation at each stage	NA
		(c) Consider use of a flow diagram	NA
Descriptive data	14*	(a) Give characteristics of study participants (eg demographic, clinical, social) and information on exposures and potential confounders	Yes
		(b) Indicate number of participants with missing data for each variable of interest	
		(c) *Cohort study*—Summarise follow-up time (eg, average and total amount)	
Outcome data	15*	*Cohort study*—Report numbers of outcome events or summary measures over time	
		Case-control study—Report numbers in each exposure category, or summary measures of exposure	
		Cross-sectional study—Report numbers of outcome events or summary measures	*Yes*
Main results	16	(*a*) Give unadjusted estimates and, if applicable, confounder-adjusted estimates and their precision (eg, 95% confidence interval). Make clear which confounders were adjusted for and why they were included	Yes
		(*b*) Report category boundaries when continuous variables were categorized	
		(*c*) If relevant, consider translating estimates of relative risk into absolute risk for a meaningful time period	
Other analyses	17	Report other analyses done—eg analyses of subgroups and interactions, and sensitivity analyses	Yes
**Discussion**			
Key results	18	Summarise key results with reference to study objectives	Yes
Limitations	19	Discuss limitations of the study, taking into account sources of potential bias or imprecision. Discuss both direction and magnitude of any potential bias	Yes
Interpretation	20	Give a cautious overall interpretation of	Yes

For peer review only - http://bmjopen.bmj.com/site/about/guidelines.xhtml
For peer review only

		results considering objectives, limitations, multiplicity of analyses, results from similar studies, and other relevant evidence	
Generalisability	21	Discuss the generalisability (external validity) of the study results	Yes
**Other information**			
Funding	22	Give the source of funding and the role of the funders for the present study and, if applicable, for the original study on which the present article is based	Yes

*Give information separately for cases and controls in case-control studies and, if applicable, for exposed and unexposed groups in cohort and cross-sectional studies.

Note: An Explanation and Elaboration article discusses each checklist item and gives methodological background and published examples of transparent reporting. The STROBE checklist is best used in conjunction with this article (freely available on the Web sites of PLoS Medicine at http://www.plosmedicine.org/, Annals of Internal Medicine at http://www.annals.org/, and Epidemiology at http://www.epidem.com/). Information on the STROBE Initiative is available at www.strobe-statement.org.

For peer review only

BMJ Open

Socio-economic inequalities in dental caries and their determinants in adolescents in New Delhi, India

Journal:	*BMJ Open*
Manuscript ID:	bmjopen-2014-006391.R1
Article Type:	Research
Date Submitted by the Author:	10-Nov-2014

Complete List of Authors:	Mathur, Manu; Public Health Foundation of India, Health Promotion<br>Tsakos, Georgios; University College London, Department of Epidemiology and Public Health<br>Millett, Christopher; Imperial College London, Primary Care and Public Health<br>Arora, Monika; Public Health Foundation of India, Health Promotion and Tobacco Control<br>Watt, Richard; University College London, Department of Epidemiology and Public Health

**Primary Subject Heading**:	Dentistry and oral medicine
Secondary Subject Heading:	Dentistry and oral medicine, Public health
Keywords:	PUBLIC HEALTH, EPIDEMIOLOGY, SOCIAL MEDICINE

SCHOLARONE™
Manuscripts

Title:

Socio-economic inequalities in dental caries and their determinants in adolescents in New Delhi, India

First and Corresponding Author:

1. **Dr. Manu Raj Mathur**

Public Health Foundation of India

ISID Campus, 4, Institutional Area, Vasant Kunj, New Delhi 110070, India

E Mail: manu.mathur@phfi.org ; Tel: +91-11-49566000

Other Authors:

2. **Dr. Georgios Tsakos**

Department of Epidemiology and Public Health, University College London

1-19 Torrington Place, London, London, WC1E 7HB

E Mail: g.tsakos@phfi.org; Tel: +44-207-6791699

3. **Prof. Christopher Millett**

School of Public Health, Imperial College London

Charing Cross Campus, London W6 8RP

E Mail: c.millett@imperial.ac.uk ; Tel: +44-207-5940817

For peer review only

For peer review only - http://bmjopen.bmj.com/site/about/guidelines.xhtml

4. Dr. Monika Arora

Public Health Foundation of India

ISID Campus, 4, Institutional Area, Vasant Kunj, New Delhi 110070, India

E Mail: monika.arora@phfi.org ; Tel: +91-11-49566000

5. Prof. Richard Watt

Department of Epidemiology and Public Health, University College London

1-19 Torrington Place, London, London, WC1E 7HB

E Mail: r.watt@phfi.org; Tel: +44-207-6791699

KEYWORDS: socio-economic inequalities, dental caries, adolescents, deprivation, India

WORD COUNT: 3050 words

For peer review only

Title

Socio-economic inequalities in dental caries and their determinants in adolescents in New Delhi, India

For peer review only

ABSTRACT

Objectives: To determine whether there were socioeconomic inequalities in caries experience and decayed teeth of Indian adolescents and assess whether behavioural and psychosocial factors explained these inequalities. **Methods:** Cross sectional study of 1,386 adolescents living in 3 diverse areas of New Delhi. Caries experience and number of decayed teeth were assessed clinically and a questionnaire was used to gather socio-demographic and psychosocial data. Zero Inflated Negative Binomial regression models were used to assess the relationship between the outcomes (caries experience and decayed teeth) and area of residence, adjusting for covariates. **Results:** Significant inequalities in caries experience and number of decayed teeth were observed. Odds of an adolescent being caries free decreased by 66% (OR 0.34, 95% CI 0.23-0.49) and 70% (OR 0.30, 95% CI 0.21-0.43) in adolescents living in resettlement communities or urban slums respectively when compared with the middle class group. No difference was observed among those with caries experience/decayed teeth. Adjusting for covariates did not affect the inequalities. **Conclusions:** Area of residence appears to be a very strong and significant determinant for an adolescent being caries/decay free in India. Psychosocial and behavioural factors do not mediate the association between area of residence and oral health.

For peer review only - http://bmjopen.bmj.com/site/about/guidelines.xhtml

RESEARCH SUMMARY

We studied the presence of socioeconomic inequalities in adolescents and the various factors which influences these inequalities. We found that significant inequalities in caries experience and number of decayed teeth exist among Indian adolescents. Adjusting for behavioural, psychosocial and material factors did not attenuate these inequalities. Relative effect of deprivation on oral health inequalities can be seen only in individuals who are disease free. In order to reduce inequalities in dental caries, there is a need to intervene early and prevent the onset of dental caries.

Strengths

- Very few studies have examined the impact of absolute poverty on the oral health of adolescents in LMICs. This study addresses this gap.
- Fairly large sample size with high response rate.
- Wide array of material, psychosocial and behavioural variables tested through standardized and reliable questionnaire.

Limitations

- This is a cross-sectional study where data was collected at one point of time.
- Risk of reporting and interviewer bias.
- Measurement issues with the scales used for psychosocial variables and for studying material deprivation

For peer review only - http://bmjopen.bmj.com/site/about/guidelines.xhtml

For peer review only

INTRODUCTION

Oral diseases are among the most common chronic diseases worldwide[1]. Oral diseases not only have an impact on general health and quality of life but may also increase the risk of mortality[2]. Oral diseases are costly for health systems and individuals, especially in those from low income and deprived households [2].

There are widespread inequalities in oral health outcomes within and between different countries of the world[3]. However, most studies examining social inequalities and gradients in oral health have been conducted in high income countries with populations that generally lie above the poverty line. As such, they could not focus on whether social inequalities exist in context of absolute poverty. No study on oral health inequalities from India has considered populations from extremely deprived areas like urban slums and resettlement communities.

Different theories have highlighted various explanations of inequalities observed in general as well as oral health [4–8]. According to these, inequalities arise because of adverse material circumstances, health-affecting behaviours or due to various psychosocial factors. Although there is a considerable amount of literature in general health[9–11], there is a paucity of evidence in the dental literature examining how different behavioural, psychosocial and socio-environmental factors influence oral health inequalities.

Our study assessed the existence of socioeconomic inequalities in dental caries of adolescents living in different geographical areas and conditions in the city of New Delhi, India. We also explored the effect of material, psychosocial and behavioural determinants on these inequalities in dental caries.

For peer review only

For peer review only - http://bmjopen.bmj.com/site/about/guidelines.xhtml

METHODS

The study was carried out in National Capital Territory (NCT) of Delhi. Nearly 0.2 million people migrate to Delhi every year and the majority of these find habitation in urban slums which constitutes about 20% of the total population of Delhi[12]. Many migrants as well as the urban poor also reside in unauthorized and resettlement communities (settlements which have recently been legalised by the Government and were previously slums and are better off economically in comparison to slums).

Study Population

This cross-sectional study was conducted among adolescents aged 12-15 years living in three diverse residential areas of New Delhi reflecting their economic position: urban slums; resettlement communities; and middle and upper middle class communities.

Study Tools:

Data were collected through an interviewer-administered questionnaire and a clinical examination. The questionnaire measured material resources, neighbourhood social capital, social support, health related behaviours (alcohol and tobacco use, diet, frequency of tooth brushing) and key socio-demographic variables. The questionnaire included pre-existing questions and scales which were checked for reliability and validity in the study population during a pilot study. Material resources were assessed using National Family Health Survey (NFHS), Standard of Living Index which has been validated and used extensively to assess material deprivation in India. Social Capital was measured by a scale developed by Gage et al (2005)[13]. This scale has been adapted from Health Behaviour in School Children (HBSC) study conducted by WHO[14]. Social Support was measured by using Social Support Scale for

For peer review only - http://bmjopen.bmj.com/site/about/guidelines.xhtml

Adolescents (SSAS) developed by Seidman et al (1995)[15]. The questions to assess health-related behaviours in adolescents were derived from the WHO Health Behaviour in School Children (HBSC) survey[14].

A non-invasive clinical examination was performed. We used the DMFT (Diseased Missing & Filled Teeth) Index to measure the level of dental caries and decayed teeth[16]. The DMFT index was calculated on every adolescent using a mouth mirror and a blunt probe. A systematic and standardised approach was used to examine the teeth based upon the WHO criteria [16]. Two trained dentists including the lead investigator performed the non-invasive clinical dental examination. Examiners were mixed periodically so that no particular examiner is confined to just one particular area for data collection and both examiners are exposed to the broad population. Inter and intra examiner reliability was checked by repeating examinations on 70 adolescents (5% of the sample). Cohen's Unweighted Kappa coefficient of agreement was used to check for internal consistency. Inter and intra-examiner agreement was above 0.83 for all teeth for the DMF index.

Study Sample:

Slums and resettlement communities were identified from an official list of registered resettlement communities and urban slums. The inclusion criteria were a) communities within a radius of 25 kilometres from the research office, b) both slum and resettlement community present together as a cluster, c) have more than 500 households in each component of the cluster, and d) have a known Non-Governmental Organization working in the community and willing to participate in the research. We identified 14 slums and resettlement communities. A census was performed in each of these communities to collect demographic data.

For peer review only - http://bmjopen.bmj.com/site/about/guidelines.xhtml

For peer review only

Adolescents from middle and upper middle class households in India generally study in private schools which have English as medium of education and charge higher fees ('English Medium Schools'). These schools were targeted to obtain the desired sample of adolescents belonging to middle and upper middle class homes. Inclusion criteria for English medium schools were: a) having secondary level classes, b) present in the same vicinity as that of the low income communities in the sampling frame, c) having at least 40 pupils per class, d) being co-educational (both boys and girls).

We used multi-stage random sampling. Five slums and resettlement communities were randomly selected from the fourteen identified communities. Amongst the selected communities, all households having at least one child of 12-15 years of age were identified. Eligible households were randomly selected and approached to request adolescents to participate in the study. If a household had 2 or more eligible adolescents then all of them were invited to participate. Five English medium private schools were randomly selected from a list 48 identified schools. From the selected schools, grades which normally have children of 12-15 years of age were identified. The class teacher then assigned random numbers to eligible adolescents in order to maintain the anonymity of identity of students. Adolescents were then randomly selected from the provided list of numbers. Both the parents and adolescents signed the consent forms after having been informed about the study.

Sample size was calculated for difference in means of the two clinical outcomes (caries experience and decayed teeth) between the three different adolescent groups, with 80% power and 5% significance level. The differences in mean values of the clinical outcomes were obtained through a pilot study conducted on 150 adolescents from a setting similar to that of this study. The calculated sample size was increased by a factor of 25% to account for potential non-

For peer review only - http://bmjopen.bmj.com/site/about/guidelines.xhtml

response, and then by a factor of 1.3 to account for the effect of clustering. The estimated sample size was 1338 adolescents (446 adolescents per group).

Variables

Main explanatory variable in this study was adolescent socioeconomic position assessed through area of residence (slums, resettlement community, middle and upper middle class homes). Covariates are grouped into the following categories; a) material resources, b) neighbourhood social capital (bridging and bonding types of social capital measuring trust, norms and reciprocity in a community), c) social support and d) health related behaviours (diet, tobacco & alcohol use, brushing frequency, visit to a dentist, getting bullied & involvement in physical fight). All these covariates were significantly associated with socioeconomic position in bivariate analyses (results not shown) and they were accounted for in the multivariable models. Outcome variables were dental caries experience and prevalence of decayed teeth.

Data Analysis

Descriptive statistics were calculated to assess the frequency distributions of explanatory and outcome variables. As there were a high number of zeros (caries/ decay free teeth) in the outcomes and the variance was considerably greater than the mean, Zero Inflated Negative Binomial (ZINB) regression analysis was used to assess the association between area of residence and caries experience (DMFT) and number of carious teeth (DT) [17]. A ZINB regression generates two separate models. The first model is a logit model generated for a ‘’certain zero’’ in the outcome (i.e. no decayed, missing or filled teeth if DMFT, or no active caries if DT is the outcome), predicting whether or not an adolescent would be in this group. The second model is the negative binomial model predicting the count of those adolescents who are

For peer review only - http://bmjopen.bmj.com/site/about/guidelines.xhtml

not in the "certain zero group", that is looking at the number of decayed, missing or filled teeth among those with DMFT>0 or at the number of decayed teeth among those with DT>0 respectively. All analyses were conducted in Stata 12.

Ethical approval was granted from University College London (UCL) Research Ethics Committee and the Public Health Foundation of India (PHFI) Technical Review and Institutional Ethics Committee.

RESULTS

A response rate of 86.6% was achieved (n = 1386). There were 736 (53.1%) boys and 650 (46.9%) girls; proportions that are almost the same to the gender distribution of Delhi NCT (53.6% males and 46.4% females) [18]. Overall, 460 (33.2%) adolescents belonged to middle and upper middle class group, 462 (33.3%) were from resettlement communities and 464 (33.5%) from urban slums. Almost half (49.7%) of the clinically examined adolescents had previous caries experience. The mean DMFT was 1.36 (1.27, 1.46). Out of the 689 adolescents with caries experience, 644 had decayed teeth at the time of clinical examination (mean = 1.21; 95%CI: 1.12, 1.31).

There was a clear social gradient, with consistently greater levels of caries experience (DMFT) at each lower level of area of residence of adolescents ($p < 0.0001$). Adolescents from middle/ upper middle class homes had mean DMFT of 0.96 (95%CI: 0.82, 1.21), those from resettlement communities had a mean of 1.38 (95% CI 1.23-1.54) and those from urban slums had a mean DMFT of 1.74 (95%CI: 1.55, 1.93). Similarly, the mean number of decayed teeth was higher at each lower socioeconomic group ($p < 0.0001$). The mean number of decayed teeth in adolescents from middle/ upper middle class homes was 0.72 (0.59, 0.85), in those from resettlement

For peer review only - http://bmjopen.bmj.com/site/about/guidelines.xhtml

communities 1.34 (1.19, 1.50) and in those from urban slums 1.58 (1.40, 1.76) (Table 1, Figure 1).

Table - 1

Socioeconomic Inequalities in Caries Experience & Decayed Teeth

**Variable**	DMFT		Carious Teeth	
	Mean (95% CI)	Median*** (IQR)	Mean (95% CI)	Median*** (IQR)
**Middle/ Upper Middle Class**	0.96 (0.82-1.21)	0 (0-1)	0.72 (0.59-0.85)	0 (0-1)
**Resettlement Communities**	1.38 (1.23-1.54)	1 (0-2)	1.34 (1.19-1.50)	1 (0-2)
**Slums**	1.74 (1.55-1.93)	1 (0-3)	1.58 (1.40-1.76)	1 (0-2)

***** p< 0.001**

IQR: Inter Quartile Range

Table 2 shows the results of the ZINB regression models for caries experience. Adjustment for covariates did not have a considerable effect on the inequalities identified. Compared to the middle/upper middle class adolescents, those living in resettlement communities had a significantly lower odds of being caries-free (OR: 0.33; 95%CI: 0.23, 0.49 in the unadjusted model, OR: 0.22; 95%CI: 0.12, 0.39 in fully adjusted model), and the same was the case for those living in urban slums (OR: 0.30, 95%CI: 0.21, 0.43 in unadjusted and OR: 0.22, 95%CI: 0.11, 0.46 in fully adjusted models). In contrast, there were no differences between the three residential sites in relation to the number of teeth with caries experience (DMFT > 0).

For peer review only - http://bmjopen.bmj.com/site/about/guidelines.xhtml

Table 2

Association between area of residence and caries experience adjusting for demographic variables, health related behaviours, material resources, social support and social capital#

(N = 1386)

	Area of Residence		
	**Middle Class**	**Resettlement Community**	**Slums**
		**OR (95% CI)**	
Logit			
**Unadjusted**	1	0.34 (0.23-0.49)***	0.30 (0.21-0.43)***
**Adjusted for Age, Sex and Religion**	1	0.28 (0.19-0.41)***	0.25 (0.17-0.37)***
**Fully Adjusted for all covariates##**	1	0.22(0.12-0.39)***	0.22 (0.11-0.46)***
		**IRR (95% CI)**	
Negative Binomial			
**Unadjusted**	1	0.88 (0.73-1.05)	1.06 (0.89-1.26)
**Adjusted for Age, Sex and Religion**	1	0.91 (0.76-1.09)	1.13 (0.94-1.35)
**Fully Adjusted for all covariates##**	1	1.00 (0.76-1.34)	1.10(0.78-1.54)

# Association tested using Zero Inflated Negative Binomial Regression Method

## Fully adjusted for age, sex, religion, health related behaviours, material resources, social support and social capital

***** p< 0.001**

The respective results for decayed teeth were similar, with a significantly lower odds of being decay-free for adolescents from resettlement communities (OR: 0.25; 95% CI: 0.17-0.37) and those from urban slums (OR: 0.24; 95% CI: 0.16-0.35) compared to their more affluent counterparts. The fully adjusted odds ratios were 0.21 ((5% CI: 0.14, 0.32) for adolescents from

For peer review only - http://bmjopen.bmj.com/site/about/guidelines.xhtml

resettlement communities and urban slums in comparison to adolescents from middle/upper middle class homes. There were no significant differences between the three groups in the number of decayed teeth among those with current decay (Table 3).

Table 3

Association between area of residence and decayed teeth adjusting for demographic variables, health related behaviours, material resources, social support and social capital[#]

(N = 1386)

	Area of Residence		
	**Middle Class**	**Resettlement Community**	**Slums**
		**OR (95% CI)**	
Logit			
**Unadjusted**	1	0.25 (0.17-0.37)***	0.24 (0.16-0.35)***
**Adjusted for Age, Sex and Religion**	1	0.21 (0.14-0.31)***	0.20 (0.13-0.30)***
**Fully Adjusted for all covariates[##]**	1	0.21(0.14-0.32)***	0.21 (0.14-0.32)***
		**IRR (95% CI)**	
Negative Binomial			
**Unadjusted**	1	0.95 (0.78-1.17)	1.11(0.91-1.35)
**Adjusted for Age, Sex and Religion**	1	1.02 (0.83-1.25)	1.21 (0.99-1.47)
**Fully Adjusted for all covariates[##]**	1	1.01 (0.82-1.24)	1.19(0.97-1.46)

[#] Association tested using Zero Inflated Negative Binomial Regression Method

[##] Fully adjusted for age, sex, religion, health related behaviours, material resources, social support and social capital

***** p< 0.001**

For peer review only - http://bmjopen.bmj.com/site/about/guidelines.xhtml

DISCUSSION

We observed a monotonic gradient for the differences in caries experience and decayed teeth between adolescents living in diverse residential areas of New Delhi. Our results showed that there is a significant difference between the proportion of individuals who were caries free or individuals with decay free teeth between the three areas of residence. However, once an individual experienced caries or developed tooth decay, it did not matter as to which residential group they belonged to as there were no significant differences in the probability of having one or more carious or decayed teeth between the three groups.

The study population comprised of adolescents from areas with extreme deprivation, such as urban slums. Only two studies have looked at the socioeconomic inequalities in dental caries in India [19,20], but they did not study adolescents from extremely deprived areas. Our findings in relation to socioeconomic inequalities observed in caries experience and number of decayed teeth are similar to previous studies conducted on adolescents[21–25], and children [26–29].

We used area of residence as an indicator of socioeconomic position of adolescents. Only one previous study has looked at inequalities in dental caries using an area based measure of socioeconomic position[27]. Thomson and Mackay (2004) in their study on 9-year-old school children from New Zealand, used both area based and individual based measures of socioeconomic position and found that the inequalities in dental caries were steeper when area based measures were used to define socioeconomic position.

Adjusting for all others factors simultaneously in our study did not cause a noteworthy change in the associations, showing that the combination of different factors investigated in this study had a limited effect on the observed inequalities in dental caries. Jung et al (2011) showed that

For peer review only - http://bmjopen.bmj.com/site/about/guidelines.xhtml

behavioural (brushing frequency, diet, smoking and alcohol use) and family affluence had no influence on the socioeconomic inequalities observed for self-reported toothache, bad breath and fractured teeth in South Korean adolescents. However, when combined with psychosocial factors (perceived stress and happiness), these factors partially accounted for the inequalities in oral health[30].

We collected data on both oral hygiene related (tooth brushing frequency, dental visit frequency) and health affecting (tobacco, alcohol, involvement in physical fight, diet) behaviours to understand the effect of behaviours on socioeconomic inequalities in oral health. None of the health affecting behaviours had any significant effect on inequalities in oral health observed in our study. Most of the studies on adolescents and young children have also shown a negligible or a minor effect of health related behaviours on inequalities in oral health[24,31,32].

Material deprivation in our study was measured through the NFHS standard of living index [33]. This index was first developed in 2000 and assessed the availability of basic material things required for living by an individual. However, India has since seen rapid economic development leading to a general improvement in the standard of living. Therefore, some of the items and respective weights used in the standard of living index may not be equally relevant in the current situation. This measurement issue may partly explain why we were not able to see any effect of material deprivation on inequalities in dental caries.

Social capital is a multidimensional concept described by different authors in different ways, therefore not easily measured with only a few items [34]. Putnam (2000) in his description of social capital stressed that community participation is also an intertwined feature along with trust and norms of reciprocity and forms an important component of social capital. The social capital questionnaire[13] used in our study measured the trust and norms of reciprocity in the society but

For peer review only - http://bmjopen.bmj.com/site/about/guidelines.xhtml

did not measure the level of community participation which might be one of the reasons of not finding any effect of social capital on inequalities in caries experience and decayed teeth. While social support may be seen as bi-directional (receiving as well as giving)[36], our scale of social support[15] measured mainly the received support or support available to an adolescent and did not assess the aspect of 'giving' support to others.

Adolescents were sampled from extremely deprived urban slums and deprived resettlement areas of New Delhi thus providing a realistic reflection of oral health inequalities in urban areas. We adopted scales and questions from internationally validated questionnaires, and further tested and adapted them for use on Indian adolescent populations. We acknowledge a number of study limitations in addition to the measurement issues about material deprivation and psychosocial variables described above. We studied only social capital and social support from the vast array of psychosocial variables. There are many other psychosocial variables like stress, depression and anxiety which were not investigated. Thus the results do not fully cover the entire psychosocial pathway to oral health inequalities. Furthermore, reporting bias cannot be ruled out as adolescents who were well versed with the consent form and objectives of the study might have given responses that are either socially desirable or perceived to be "wanted" by the interviewer.

Our study findings suggest that the relative impact of deprivation on oral health inequalities is seen only in individuals who are disease free, with a clear gradient indicating higher prevalence of adolescents free from caries (or caries experience) for each consecutively less deprived area of residence. Our study has also shown that area of residence may be a very important determinant of the oral health status of adolescents in India. Psychosocial, material or behavioural characteristics did not mediate the role of extreme living conditions on oral health. This finding

For peer review only - http://bmjopen.bmj.com/site/about/guidelines.xhtml

For peer review only

highlights the importance of health promotion [37], in reducing inequalities in oral health. In order to reduce inequalities in dental caries, there is a need to intervene early and prevent the onset of dental caries and 'act before it happens' rather than intervening after caries has affected the population. There is a need to design policies which aim at primary prevention and improving health by taking action on the broader structural determinants of health.

AUTHOR'S CONTRIBUTIONS

MRM, RGW, GT and MA conceptualized and designed the study. MRM collected the data, performed analysis and wrote the manuscript. RGW and GT directed the development of methodology, analytic plan and interpretation of results. MRM, RW, GT and CM undertook the critical revisions of the paper for substantial intellectual content. MA contributed to the background and methods section of this paper. All the authors approved of the final version to be published.

COMPETING INTEREST

We declare no competing interest.

ACKNOWLEDGEMENTS/ FUNDING

This work was supported by a Wellcome Trust Capacity Strengthening Strategic Award to the Public Health Foundation of India and a consortium of UK universities. CM is funded by a NIHR Research Professorship award.

DATA SHARING

No additional data available

For peer review only

REFERENCES:

1 Petersen PE. Priorities for research for oral health in the 21st century--the approach of the WHO Global Oral Health Programme. *Community dental health* 2005;**22**:71–4.

2 Petersen PE. The World Oral Health Report 2003 WHO Global Oral Health Programme. *Oral Health* 2003;**31 Suppl 1**:3–23.

3 Marmot M. Social determinants of health inequalities. *Lancet* 2005;**365**:1099–104. doi:10.1016/S0140-6736(05)71146-6

4 Watt RG. Emerging theories into the social determinants of health: implications for oral health promotion. *Community dentistry and oral epidemiology* 2002;**30**:241–7.

5 Sisson KL. Theoretical explanations for social inequalities in oral health. *Community dentistry and oral epidemiology* 2007;**35**:81–8. doi:10.1111/j.1600-0528.2007.00354.x

6 Black D., Townsend P, Davidson N. *Inequalities in health : the Black report*. Harmondsworth: : Penguin 1980.

7 Whitehead M. *Inequalities in Health: The Black Report and the Health Divide*. Harmondsworth: : Penguin 1986.

8 Bartley M. *Health Inequality: an introduction to theories, concepts and methods*. Oxford: : Blackwell Publishing Limited 2004.

9 Karriker-Jaffe KJ, Foshee VA, Ennett ST, *et al.* Sex differences in the effects of neighborhood socioeconomic disadvantage and social organization on rural adolescents' aggression trajectories. *American journal of community psychology* 2009;**43**:189–203. doi:10.1007/s10464-009-9236-x

10 Kohen DE, Leventhal T, Dahinten VS, *et al.* Neighborhood disadvantage: pathways of effects for young children. *Child development* 2008;**79**:156–69. doi:10.1111/j.1467-8624.2007.01117.x

11 Sabbah W, Watt RG, Sheiham A, *et al.* The role of cognitive ability in socio-economic inequalities in oral health. *Journal of Dental Research* 2009;**88**:351–5.

12 MoHFW. STATE OF URBAN HEALTH IN DELHI. New Delhi: 2007.

13 Gage JC, Overpeck MD, Nansel TR, *et al.* Peer activity in the evenings and participation in aggressive and problem behaviors. *The Journal of adolescent health : official publication of the Society for Adolescent Medicine* 2005;**37**:517. doi:10.1016/j.jadohealth.2004.12.012

For peer review only - http://bmjopen.bmj.com/site/about/guidelines.xhtml

14 Roberts C, Freeman J, Samdal O, *et al.* The Health Behaviour in School-aged Children (HBSC) study: methodological developments and current tensions. *International journal of public health* 2009;**54 Suppl 2**:140–50. doi:10.1007/s00038-009-5405-9

15 Seidman E, Allen L, Aber JL, *et al.* Development and validation of adolescent-perceived microsystem scales: social support, daily hassles, and involvement. *American journal of community psychology* 1995;**23**:355–88.

16 WHO. *Oral Health Surveys; Basic Methods*. 4th Editio. Geneva: : World Health Organization 1997.

17 Lewsey JD, Thomson WM. The utility of the zero-inflated Poisson and zero-inflated negative binomial models: a case study of cross-sectional and longitudinal DMF data examining the effect of socio-economic status. *Community dentistry and oral epidemiology* 2004;**32**:183–9. doi:10.1111/j.1600-0528.2004.00155.x

18 Census of India. Tables and Statements. 2011.http://www.censusindia.gov.in/2011-prov-results/data_files/delhi/3_PDFC-Paper-1-tables_60_81.pdf (accessed 3 Nov2012).

19 Christian B, Evans RW. Has urbanization become a risk factor for dental caries in Kerala, India: a cross-sectional study of children aged 6 and 12 years. *International journal of paediatric dentistry* 2009;**19**:330–7. doi:10.1111/j.1365-263X.2009.00979.x

20 Singh A, Sequiera P, Acharya S, *et al.* Oral health status of two 12-year-old socially disadvantaged groups in South India: a comparative study. *Oral health & preventive dentistry* 2011;**9**:3–7.

21 Armfield JM. Socioeconomic inequalities in child oral health: a comparison of discrete and composite area-based measures. *Journal of public health dentistry* 2007;**67**:119–25.

22 Freire MC, Sheiham A, Hardy R. Adolescents' sense of coherence, oral health status, and oral health-related behaviours. *Community Dentistry & Oral Epidemiology* 2001;**29**:204–12.

23 Perera I, Ekanayake L. Factors influencing perception of oral health among adolescents in Sri Lanka. *International Dental Journal* 2008;**58**:349–55.

24 Polk DE, Weyant RJ, Manz MC. Socioeconomic factors in adolescents' oral health: are they mediated by oral hygiene behaviors or preventive interventions? *Community dentistry and oral epidemiology* 2010;**38**:1–9. doi:10.1111/j.1600-0528.2009.00499.x

25 Zurriaga O, Martíínez-Beneito MA, Abellán JJ, *et al.* Assessing the social class of children from parental information to study possible social inequalities in health outcomes. *Annals of epidemiology* 2004;**14**:378–84. doi:10.1016/j.annepidem.2003.06.001

For peer review only

For peer review only - http://bmjopen.bmj.com/site/about/guidelines.xhtml

26 Poulton R, Caspi A, Milne BJ, *et al.* Association between children's experience of socioeconomic disadvantage and adult health: a life-course study. *Lancet* 2002;**360**:1640–5. doi:10.1016/S0140-6736(02)11602-3

27 Thomson WM, Mackay TD. Child dental caries patterns described using a combination of area-based and household-based socio-economic status measures. *Community Dental Health* 2004;**21**:285–90.

28 Sagheri D, McLoughlin J, Clarkson JJ. The prevalence of dental caries and fissure sealants in 12 year old children by disadvantaged status in Dublin (Ireland). *Community Dental Health* 2009;**26**:32–7.

29 Van den Branden S, Van den Broucke S, Leroy R, *et al.* Effects of time and socio-economic status on the determinants of oral health-related behaviours of parents of preschool children. *European Journal of Oral Sciences* 2012;**120**:153–60.

30 Jung SH, Watt RG, Sheiham A, *et al.* Exploring pathways for socioeconomic inequalities in self-reported oral symptoms among Korean adolescents. *Community Dentistry and Oral Epidemiology* 2011;**39**:221–9.

31 Ravaghi V, Underwood M, Marinho V, *et al.* Socioeconomic status and self-reported oral health in Iranian adolescents: The role of selected oral health behaviors and psychological factors. *Journal of Public Health Dentistry* Published Online First: 2012.

32 Sanders AE, Spencer a J, Slade GD. Evaluating the role of dental behaviour in oral health inequalities. *Community dentistry and oral epidemiology* 2006;**34**:71–9. doi:10.1111/j.1600-0528.2006.00261.x

33 Vyas S, Kumaranayake L. Constructing socio-economic status indices: how to use principal components analysis. *Health policy and planning* 2006;**21**:459–68. doi:10.1093/heapol/czl029

34 Berry HL, Welsh JA. Social capital and health in Australia: An overview from the household, income and labour dynamics in Australia survey. *Social science & medicine (1982)* 2010;**70**:588–96. doi:10.1016/j.socscimed.2009.10.012

35 Putnam R. *Bowling Alone: The Collapse and Revival of American Community*. New York: : Simon and Schuster 2000.

36 Kahn RL, Antonucci TC. Convoys over the lifecourse; attachment roles and social support. In: Baltes PB, Brim O, eds. *Life span development and behaviour*. New York: : Academic Press 1980. 253–86.

37 WHO. The Ottawa Charter for Health Promotion. Geneva: 1986.

For peer review only

For peer review only - http://bmjopen.bmj.com/site/about/guidelines.xhtml

Title:

Socio-economic inequalities in dental caries and their determinants in adolescents in New Delhi, India

First and Corresponding Author:

1. **Dr. Manu Raj Mathur**

Public Health Foundation of India

ISID Campus, 4, Institutional Area, Vasant Kunj, New Delhi 110070, India

E Mail: manu.mathur@phfi.org ; Tel: +91-11-49566000

Other Authors:

2. **Dr. Georgios Tsakos**

Department of Epidemiology and Public Health, University College London

1-19 Torrington Place, London, London, WC1E 7HB

E Mail: g.tsakos@phfi.org; Tel: +44-207-6791699

3. **Prof. Christopher Millett**

School of Public Health, Imperial College London

Charing Cross Campus, London W6 8RP

E Mail: c.millett@imperial.ac.uk ; Tel: +44-207-5940817

For peer review only - http://bmjopen.bmj.com/site/about/guidelines.xhtml

4. Dr. Monika Arora

Public Health Foundation of India

ISID Campus, 4, Institutional Area, Vasant Kunj, New Delhi 110070, India

E Mail: monika.arora@phfi.org ; Tel: +91-11-49566000

5. Prof. Richard Watt

Department of Epidemiology and Public Health, University College London

1-19 Torrington Place, London, London, WC1E 7HB

E Mail: r.watt@phfi.org; Tel: +44-207-6791699

KEYWORDS: socio-economic inequalities, dental caries, adolescents, deprivation, India

WORD COUNT: 3050 words

For peer review only - http://bmjopen.bmj.com/site/about/guidelines.xhtml

Title

Socio-economic inequalities in dental caries and their determinants in adolescents in New Delhi, India

ABSTRACT

Objectives: To determine whether there were socioeconomic inequalities in caries experience and decayed teeth of Indian adolescents and assess whether behavioural and psychosocial factors explained these inequalities. **Methods:** Cross sectional study of 1,386 adolescents living in 3 diverse areas of New Delhi. Caries experience and number of decayed teeth were assessed clinically and a questionnaire was used to gather socio-demographic and psychosocial data. Zero Inflated Negative Binomial regression models were used to assess the relationship between the outcomes (caries experience and decayed teeth) and area of residence, adjusting for covariates. **Results:** Significant inequalities in caries experience and number of decayed teeth were observed. Odds of an adolescent being caries free decreased by 66% (OR 0.34, 95% CI 0.23-0.49) and 70% (OR 0.30, 95% CI 0.21-0.43) in adolescents living in resettlement communities or urban slums respectively when compared with the middle class group. No difference was observed among those with caries experience/decayed teeth. Adjusting for covariates did not affect the inequalities. **Conclusions:** Area of residence appears to be a very strong and significant determinant for an adolescent being caries/decay free in India. Psychosocial and behavioural factors do not mediate the association between area of residence and oral health.

For peer review only - http://bmjopen.bmj.com/site/about/guidelines.xhtml

RESEARCH SUMMARY

We studied the presence of socioeconomic inequalities in adolescents and the various factors which influences these inequalities. We found that significant inequalities in caries experience and number of decayed teeth exist among Indian adolescents. Adjusting for behavioural, psychosocial and material factors did not attenuate these inequalities. Relative effect of deprivation on oral health inequalities can be seen only in individuals who are disease free. In order to reduce inequalities in dental caries, there is a need to intervene early and prevent the onset of dental caries.

Strengths

- Very few studies have examined the impact of absolute poverty on the oral health of adolescents in LMICs. This study addresses this gap.
- Fairly large sample size with high response rate.
- Wide array of material, psychosocial and behavioural variables tested through standardized and reliable questionnaire.

Limitations

- This is a cross-sectional study where data was collected at one point of time.
- Risk of reporting and interviewer bias.
- Measurement issues with the scales used for psychosocial variables and for studying material deprivation

For peer review only

INTRODUCTION

Oral diseases are among the most common chronic diseases worldwide[1]. Oral diseases not only have an impact on general health and quality of life but may also increase the risk of mortality[2]. Oral diseases are costly for health systems and individuals, especially in those from low income and deprived households [2].

There are widespread inequalities in oral health outcomes within and between different countries of the world[3]. However, most studies examining social inequalities and gradients in oral health have been conducted in high income countries with populations that generally lie above the poverty line. As such, they could not focus on whether social inequalities exist in context of absolute poverty. No study on oral health inequalities from India has considered populations from extremely deprived areas like urban slums and resettlement communities.

Different theories have highlighted various explanations of inequalities observed in general as well as oral health [4–8]. According to these, inequalities arise because of adverse material circumstances, health-affecting behaviours or due to various psychosocial factors. Although there is a considerable amount of literature in general health[9–11], there is a paucity of evidence in the dental literature examining how different behavioural, psychosocial and socio-environmental factors influence oral health inequalities.

Our study assessed the existence of socioeconomic inequalities in dental caries of adolescents living in different geographical areas and conditions in the city of New Delhi, India. We also explored the effect of material, psychosocial and behavioural determinants on these inequalities in dental caries.

For peer review only

METHODS

The study was carried out in National Capital Territory (NCT) of Delhi. Nearly 0.2 million people migrate to Delhi every year and the majority of these find habitation in urban slums which constitutes about 20% of the total population of Delhi[12]. Many migrants as well as the urban poor also reside in unauthorized and resettlement communities (settlements which have recently been legalised by the Government and were previously slums and are better off economically in comparison to slums).

Study Population

This cross-sectional study was conducted among adolescents aged 12-15 years living in three diverse residential areas of New Delhi reflecting their economic position: urban slums; resettlement communities; and middle and upper middle class communities.

Study Tools:

Data were collected through an interviewer-administered questionnaire and a clinical examination. The questionnaire measured material resources, neighbourhood social capital, social support, health related behaviours (alcohol and tobacco use, diet, frequency of tooth brushing) and key socio-demographic variables. The questionnaire included pre-existing questions and scales which were checked for reliability and validity in the study population during a pilot study. Material resources were assessed using National Family Health Survey (NFHS), Standard of Living Index which has been validated and used extensively to assess material deprivation in India. Social Capital was measured by a scale developed by Gage et al (2005)[13]. This scale has been adapted from Health Behaviour in School Children (HBSC) study conducted by WHO[14]. Social Support was measured by using Social Support Scale for

For peer review only - http://bmjopen.bmj.com/site/about/guidelines.xhtml

For peer review only

Adolescents (SSAS) developed by Seidman et al (1995)[15]. The questions to assess health-related behaviours in adolescents were derived from the WHO Health Behaviour in School Children (HBSC) survey[14].

A non-invasive clinical examination was performed. We used the DMFT (Diseased Missing & Filled Teeth) Index to measure the level of dental caries and decayed teeth[16]. The DMFT index was calculated on every adolescent using a mouth mirror and a blunt probe. A systematic and standardised approach was used to examine the teeth based upon the WHO criteria [16]. Two trained dentists including the lead investigator performed the non-invasive clinical dental examination. Examiners were mixed periodically so that no particular examiner is confined to just one particular area for data collection and both examiners are exposed to the broad population. Inter and intra examiner reliability was checked by repeating examinations on 70 adolescents (5% of the sample). Cohen's Unweighted Kappa coefficient of agreement was used to check for internal consistency. Inter and intra-examiner agreement was above 0.83 for all teeth for the DMF index.

Study Sample:

Slums and resettlement communities were identified from an official list of registered resettlement communities and urban slums. The inclusion criteria were a) communities within a radius of 25 kilometres from the research office, b) both slum and resettlement community present together as a cluster, c) have more than 500 households in each component of the cluster, and d) have a known Non-Governmental Organization working in the community and willing to participate in the research. We identified 14 slums and resettlement communities. A census was performed in each of these communities to collect demographic data.

For peer review only - http://bmjopen.bmj.com/site/about/guidelines.xhtml

Adolescents from middle and upper middle class households in India generally study in private schools which have English as medium of education and charge higher fees (‘English Medium Schools’). These schools were targeted to obtain the desired sample of adolescents belonging to middle and upper middle class homes. Inclusion criteria for English medium schools were: a) having secondary level classes, b) present in the same vicinity as that of the low income communities in the sampling frame, c) having at least 40 pupils per class, d) being co-educational (both boys and girls).

We used multi-stage random sampling. Five slums and resettlement communities were randomly selected from the fourteen identified communities. Amongst the selected communities, all households having at least one child of 12-15 years of age were identified. Eligible households were randomly selected and approached to request adolescents to participate in the study. If a household had 2 or more eligible adolescents then all of them were invited to participate. Five English medium private schools were randomly selected from a list 48 identified schools. From the selected schools, grades which normally have children of 12-15 years of age were identified. The class teacher then assigned random numbers to eligible adolescents in order to maintain the anonymity of identity of students. Adolescents were then randomly selected from the provided list of numbers. Both the parents and adolescents signed the consent forms after having been informed about the study.

Sample size was calculated for difference in means of the two clinical outcomes (caries experience and decayed teeth) between the three different adolescent groups, with 80% power and 5% significance level. The differences in mean values of the clinical outcomes were obtained through a pilot study conducted on 150 adolescents from a setting similar to that of this study. The calculated sample size was increased by a factor of 25% to account for potential non-

For peer review only - http://bmjopen.bmj.com/site/about/guidelines.xhtml

response, and then by a factor of 1.3 to account for the effect of clustering. The estimated sample size was 1338 adolescents (446 adolescents per group).

Variables

Main explanatory variable in this study was adolescent socioeconomic position assessed through area of residence (slums, resettlement community, middle and upper middle class homes). Covariates are grouped into the following categories; a) material resources, b) neighbourhood social capital (bridging and bonding types of social capital measuring trust, norms and reciprocity in a community), c) social support and d) health related behaviours (diet, tobacco & alcohol use, brushing frequency, visit to a dentist, getting bullied & involvement in physical fight). All these covariates were significantly associated with socioeconomic position in bivariate analyses (results not shown) and they were accounted for in the multivariable models. Outcome variables were dental caries experience and prevalence of decayed teeth.

Data Analysis

Descriptive statistics were calculated to assess the frequency distributions of explanatory and outcome variables. As there were a high number of zeros (caries/ decay free teeth) in the outcomes and the variance was considerably greater than the mean, Zero Inflated Negative Binomial (ZINB) regression analysis was used to assess the association between area of residence and caries experience (DMFT) and number of carious teeth (DT) [17]. A ZINB regression generates two separate models. The first model is a logit model generated for a ‘’certain zero’’ in the outcome (i.e. no decayed, missing or filled teeth if DMFT, or no active caries if DT is the outcome), predicting whether or not an adolescent would be in this group. The second model is the negative binomial model predicting the count of those adolescents who are

For peer review only - http://bmjopen.bmj.com/site/about/guidelines.xhtml

not in the “certain zero group”, that is looking at the number of decayed, missing or filled teeth among those with DMFT>0 or at the number of decayed teeth among those with DT>0 respectively. All analyses were conducted in Stata 12.

Ethical approval was granted from University College London (UCL) Research Ethics Committee and the Public Health Foundation of India (PHFI) Technical Review and Institutional Ethics Committee.

RESULTS

A response rate of 86.6% was achieved (n = 1386). There were 736 (53.1%) boys and 650 (46.9%) girls; proportions that are almost the same to the gender distribution of Delhi NCT (53.6% males and 46.4% females) [18]. Overall, 460 (33.2%) adolescents belonged to middle and upper middle class group, 462 (33.3%) were from resettlement communities and 464 (33.5%) from urban slums. Almost half (49.7%) of the clinically examined adolescents had previous caries experience. The mean DMFT was 1.36 (1.27, 1.46). Out of the 689 adolescents with caries experience, 644 had decayed teeth at the time of clinical examination (mean = 1.21; 95%CI: 1.12, 1.31).

There was a clear social gradient, with consistently greater levels of caries experience (DMFT) at each lower level of area of residence of adolescents ($p < 0.0001$). Adolescents from middle/ upper middle class homes had mean DMFT of 0.96 (95%CI: 0.82, 1.21), those from resettlement communities had a mean of 1.38 (95% CI 1.23-1.54) and those from urban slums had a mean DMFT of 1.74 (95%CI: 1.55, 1.93). Similarly, the mean number of decayed teeth was higher at each lower socioeconomic group ($p < 0.0001$). The mean number of decayed teeth in adolescents from middle/ upper middle class homes was 0.72 (0.59, 0.85), in those from resettlement

For peer review only - http://bmjopen.bmj.com/site/about/guidelines.xhtml

communities 1.34 (1.19, 1.50) and in those from urban slums 1.58 (1.40, 1.76) (Table 1, Figure 1).

Table - 1

Socioeconomic Inequalities in Caries Experience & Decayed Teeth

**Variable**	DMFT		Carious Teeth	
	Mean (95% CI)	Median*** (IQR)	Mean (95% CI)	Median*** (IQR)
**Middle/ Upper Middle Class**	0.96 (0.82-1.21)	0 (0-1)	0.72 (0.59-0.85)	0 (0-1)
**Resettlement Communities**	1.38 (1.23-1.54)	1 (0-2)	1.34 (1.19-1.50)	1 (0-2)
**Slums**	1.74 (1.55-1.93)	1 (0-3)	1.58 (1.40-1.76)	1 (0-2)

***** p< 0.001**

IQR: Inter Quartile Range

Table 2 shows the results of the ZINB regression models for caries experience. Adjustment for covariates did not have a considerable effect on the inequalities identified. Compared to the middle/upper middle class adolescents, those living in resettlement communities had a significantly lower odds of being caries-free (OR: 0.33; 95%CI: 0.23, 0.49 in the unadjusted model, OR: 0.22; 95%CI: 0.12, 0.39 in fully adjusted model), and the same was the case for those living in urban slums (OR: 0.30, 95%CI: 0.21, 0.43 in unadjusted and OR: 0.22, 95%CI: 0.11, 0.46 in fully adjusted models). In contrast, there were no differences between the three residential sites in relation to the number of teeth with caries experience (DMFT > 0).

For peer review only - http://bmjopen.bmj.com/site/about/guidelines.xhtml

Table 2

Association between area of residence and caries experience adjusting for demographic variables, health related behaviours, material resources, social support and social capital[#]

(N = 1386)

	Area of Residence		
	**Middle Class**	**Resettlement Community**	**Slums**
		**OR (95% CI)**	
Logit			
**Unadjusted**	1	0.34 (0.23-0.49)***	0.30 (0.21-0.43)***
**Adjusted for Age, Sex and Religion**	1	0.28 (0.19-0.41)***	0.25 (0.17-0.37)***
**Fully Adjusted for all covariates[##]**	1	0.22(0.12-0.39)***	0.22 (0.11-0.46)***
		**IRR (95% CI)**	
Negative Binomial			
**Unadjusted**	1	0.88 (0.73-1.05)	1.06 (0.89-1.26)
**Adjusted for Age, Sex and Religion**	1	0.91 (0.76-1.09)	1.13 (0.94-1.35)
**Fully Adjusted for all covariates[##]**	1	1.00 (0.76-1.34)	1.10(0.78-1.54)

[#] Association tested using Zero Inflated Negative Binomial Regression Method

[##] Fully adjusted for age, sex, religion, health related behaviours, material resources, social support and social capital

***** p< 0.001**

The respective results for decayed teeth were similar, with a significantly lower odds of being decay-free for adolescents from resettlement communities (OR: 0.25; 95% CI: 0.17-0.37) and those from urban slums (OR: 0.24; 95% CI: 0.16-0.35) compared to their more affluent counterparts. The fully adjusted odds ratios were 0.21 ((5% CI: 0.14, 0.32) for adolescents from

For peer review only - http://bmjopen.bmj.com/site/about/guidelines.xhtml

resettlement communities and urban slums in comparison to adolescents from middle/upper middle class homes. There were no significant differences between the three groups in the number of decayed teeth among those with current decay (Table 3).

Table 3

Association between area of residence and decayed teeth adjusting for demographic variables, health related behaviours, material resources, social support and social capital[#]

(N = 1386)

	Area of Residence		
	**Middle Class**	**Resettlement Community**	**Slums**
		**OR (95% CI)**	
Logit			
**Unadjusted**	1	0.25 (0.17-0.37)***	0.24 (0.16-0.35)***
**Adjusted for Age, Sex and Religion**	1	0.21 (0.14-0.31)***	0.20 (0.13-0.30)***
**Fully Adjusted for all covariates[##]**	1	0.21(0.14-0.32)***	0.21 (0.14-0.32)***
		**IRR (95% CI)**	
Negative Binomial			
**Unadjusted**	1	0.95 (0.78-1.17)	1.11(0.91-1.35)
**Adjusted for Age, Sex and Religion**	1	1.02 (0.83-1.25)	1.21 (0.99-1.47)
**Fully Adjusted for all covariates[##]**	1	1.01 (0.82-1.24)	1.19(0.97-1.46)

[#] **Association tested using Zero Inflated Negative Binomial Regression Method**

[##] **Fully adjusted for age, sex, religion, health related behaviours, material resources, social support and social capital**

***** p< 0.001**

For peer review only

For peer review only - http://bmjopen.bmj.com/site/about/guidelines.xhtml

DISCUSSION

We observed a monotonic gradient for the differences in caries experience and decayed teeth between adolescents living in diverse residential areas of New Delhi. Our results showed that there is a significant difference between the proportion of individuals who were caries free or individuals with decay free teeth between the three areas of residence. However, once an individual experienced caries or developed tooth decay, it did not matter as to which residential group they belonged to as there were no significant differences in the probability of having one or more carious or decayed teeth between the three groups.

The study population comprised of adolescents from areas with extreme deprivation, such as urban slums. Only two studies have looked at the socioeconomic inequalities in dental caries in India [19,20], but they did not study adolescents from extremely deprived areas. Our findings in relation to socioeconomic inequalities observed in caries experience and number of decayed teeth are similar to previous studies conducted on adolescents[21–25], and children [26–29].

We used area of residence as an indicator of socioeconomic position of adolescents. Only one previous study has looked at inequalities in dental caries using an area based measure of socioeconomic position[27]. Thomson and Mackay (2004) in their study on 9-year-old school children from New Zealand, used both area based and individual based measures of socioeconomic position and found that the inequalities in dental caries were steeper when area based measures were used to define socioeconomic position.

Adjusting for all others factors simultaneously in our study did not cause a noteworthy change in the associations, showing that the combination of different factors investigated in this study had a limited effect on the observed inequalities in dental caries. Jung et al (2011) showed that

For peer review only

behavioural (brushing frequency, diet, smoking and alcohol use) and family affluence had no influence on the socioeconomic inequalities observed for self-reported toothache, bad breath and fractured teeth in South Korean adolescents. However, when combined with psychosocial factors (perceived stress and happiness), these factors partially accounted for the inequalities in oral health[30].

We collected data on both oral hygiene related (tooth brushing frequency, dental visit frequency) and health affecting (tobacco, alcohol, involvement in physical fight, diet) behaviours to understand the effect of behaviours on socioeconomic inequalities in oral health. None of the health affecting behaviours had any significant effect on inequalities in oral health observed in our study. Most of the studies on adolescents and young children have also shown a negligible or a minor effect of health related behaviours on inequalities in oral health[24,31,32].

Material deprivation in our study was measured through the NFHS standard of living index [33]. This index was first developed in 2000 and assessed the availability of basic material things required for living by an individual. However, India has since seen rapid economic development leading to a general improvement in the standard of living. Therefore, some of the items and respective weights used in the standard of living index may not be equally relevant in the current situation. This measurement issue may partly explain why we were not able to see any effect of material deprivation on inequalities in dental caries.

Social capital is a multidimensional concept described by different authors in different ways, therefore not easily measured with only a few items [34]. Putnam (2000) in his description of social capital stressed that community participation is also an intertwined feature along with trust and norms of reciprocity and forms an important component of social capital. The social capital questionnaire[13] used in our study measured the trust and norms of reciprocity in the society but

For peer review only - http://bmjopen.bmj.com/site/about/guidelines.xhtml

did not measure the level of community participation which might be one of the reasons of not finding any effect of social capital on inequalities in caries experience and decayed teeth. While social support may be seen as bi-directional (receiving as well as giving)[36], our scale of social support[15] measured mainly the received support or support available to an adolescent and did not assess the aspect of 'giving' support to others.

Adolescents were sampled from extremely deprived urban slums and deprived resettlement areas of New Delhi thus providing a realistic reflection of oral health inequalities in urban areas. We adopted scales and questions from internationally validated questionnaires, and further tested and adapted them for use on Indian adolescent populations. We acknowledge a number of study limitations in addition to the measurement issues about material deprivation and psychosocial variables described above. We studied only social capital and social support from the vast array of psychosocial variables. There are many other psychosocial variables like stress, depression and anxiety which were not investigated. Thus the results do not fully cover the entire psychosocial pathway to oral health inequalities. Furthermore, reporting bias cannot be ruled out as adolescents who were well versed with the consent form and objectives of the study might have given responses that are either socially desirable or perceived to be "wanted" by the interviewer.

Our study findings suggest that the relative impact of deprivation on oral health inequalities is seen only in individuals who are disease free, with a clear gradient indicating higher prevalence of adolescents free from caries (or caries experience) for each consecutively less deprived area of residence. Our study has also shown that area of residence may be a very important determinant of the oral health status of adolescents in India. Psychosocial, material or behavioural characteristics did not mediate the role of extreme living conditions on oral health. This finding

For peer review only - http://bmjopen.bmj.com/site/about/guidelines.xhtml

highlights the importance of health promotion [37], in reducing inequalities in oral health. In order to reduce inequalities in dental caries, there is a need to intervene early and prevent the onset of dental caries and 'act before it happens' rather than intervening after caries has affected the population. There is a need to design policies which aim at primary prevention and improving health by taking action on the broader structural determinants of health.

AUTHOR'S CONTRIBUTIONS

MRM, RGW, GT and MA conceptualized and designed the study. MRM collected the data, performed analysis and wrote the manuscript. RGW and GT directed the development of methodology, analytic plan and interpretation of results. MRM, RW, GT and CM undertook the critical revisions of the paper for substantial intellectual content. MA contributed to the background and methods section of this paper. All the authors approved of the final version to be published.

COMPETING INTEREST

We declare no competing interest.

ACKNOWLEDGEMENTS/ FUNDING

This work was supported by a Wellcome Trust Capacity Strengthening Strategic Award to the Public Health Foundation of India and a consortium of UK universities. CM is funded by a NIHR Research Professorship award.

DATA SHARING

No additional data available

For peer review only - http://bmjopen.bmj.com/site/about/guidelines.xhtml

For peer review only

REFERENCES:

1 Petersen PE. Priorities for research for oral health in the 21st century--the approach of the WHO Global Oral Health Programme. *Community dental health* 2005;**22**:71–4.

2 Petersen PE. The World Oral Health Report 2003 WHO Global Oral Health Programme. *Oral Health* 2003;**31 Suppl 1**:3–23.

3 Marmot M. Social determinants of health inequalities. *Lancet* 2005;**365**:1099–104. doi:10.1016/S0140-6736(05)71146-6

4 Watt RG. Emerging theories into the social determinants of health: implications for oral health promotion. *Community dentistry and oral epidemiology* 2002;**30**:241–7.

5 Sisson KL. Theoretical explanations for social inequalities in oral health. *Community dentistry and oral epidemiology* 2007;**35**:81–8. doi:10.1111/j.1600-0528.2007.00354.x

6 Black D., Townsend P, Davidson N. *Inequalities in health : the Black report*. Harmondsworth: : Penguin 1980.

7 Whitehead M. *Inequalities in Health: The Black Report and the Health Divide*. Harmondsworth: : Penguin 1986.

8 Bartley M. *Health Inequality: an introduction to theories, concepts and methods*. Oxford: : Blackwell Publishing Limited 2004.

9 Karriker-Jaffe KJ, Foshee VA, Ennett ST, *et al.* Sex differences in the effects of neighborhood socioeconomic disadvantage and social organization on rural adolescents' aggression trajectories. *American journal of community psychology* 2009;**43**:189–203. doi:10.1007/s10464-009-9236-x

10 Kohen DE, Leventhal T, Dahinten VS, *et al.* Neighborhood disadvantage: pathways of effects for young children. *Child development* 2008;**79**:156–69. doi:10.1111/j.1467-8624.2007.01117.x

11 Sabbah W, Watt RG, Sheiham A, *et al.* The role of cognitive ability in socio-economic inequalities in oral health. *Journal of Dental Research* 2009;**88**:351–5.

12 MoHFW. STATE OF URBAN HEALTH IN DELHI. New Delhi: 2007.

13 Gage JC, Overpeck MD, Nansel TR, *et al.* Peer activity in the evenings and participation in aggressive and problem behaviors. *The Journal of adolescent health : official publication of the Society for Adolescent Medicine* 2005;**37**:517. doi:10.1016/j.jadohealth.2004.12.012

For peer review only - http://bmjopen.bmj.com/site/about/guidelines.xhtml

14 Roberts C, Freeman J, Samdal O, *et al.* The Health Behaviour in School-aged Children (HBSC) study: methodological developments and current tensions. *International journal of public health* 2009;**54 Suppl 2**:140–50. doi:10.1007/s00038-009-5405-9

15 Seidman E, Allen L, Aber JL, *et al.* Development and validation of adolescent-perceived microsystem scales: social support, daily hassles, and involvement. *American journal of community psychology* 1995;**23**:355–88.

16 WHO. *Oral Health Surveys; Basic Methods*. 4th Editio. Geneva: : World Health Organization 1997.

17 Lewsey JD, Thomson WM. The utility of the zero-inflated Poisson and zero-inflated negative binomial models: a case study of cross-sectional and longitudinal DMF data examining the effect of socio-economic status. *Community dentistry and oral epidemiology* 2004;**32**:183–9. doi:10.1111/j.1600-0528.2004.00155.x

18 Census of India. Tables and Statements. 2011.http://www.censusindia.gov.in/2011-prov-results/data_files/delhi/3_PDFC-Paper-1-tables_60_81.pdf (accessed 3 Nov2012).

19 Christian B, Evans RW. Has urbanization become a risk factor for dental caries in Kerala, India: a cross-sectional study of children aged 6 and 12 years. *International journal of paediatric dentistry* 2009;**19**:330–7. doi:10.1111/j.1365-263X.2009.00979.x

20 Singh A, Sequiera P, Acharya S, *et al.* Oral health status of two 12-year-old socially disadvantaged groups in South India: a comparative study. *Oral health & preventive dentistry* 2011;**9**:3–7.

21 Armfield JM. Socioeconomic inequalities in child oral health: a comparison of discrete and composite area-based measures. *Journal of public health dentistry* 2007;**67**:119–25.

22 Freire MC, Sheiham A, Hardy R. Adolescents' sense of coherence, oral health status, and oral health-related behaviours. *Community Dentistry & Oral Epidemiology* 2001;**29**:204–12.

23 Perera I, Ekanayake L. Factors influencing perception of oral health among adolescents in Sri Lanka. *International Dental Journal* 2008;**58**:349–55.

24 Polk DE, Weyant RJ, Manz MC. Socioeconomic factors in adolescents' oral health: are they mediated by oral hygiene behaviors or preventive interventions? *Community dentistry and oral epidemiology* 2010;**38**:1–9. doi:10.1111/j.1600-0528.2009.00499.x

25 Zurriaga O, Martíínez-Beneito MA, Abellán JJ, *et al.* Assessing the social class of children from parental information to study possible social inequalities in health outcomes. *Annals of epidemiology* 2004;**14**:378–84. doi:10.1016/j.annepidem.2003.06.001

For peer review only - http://bmjopen.bmj.com/site/about/guidelines.xhtml

26 Poulton R, Caspi A, Milne BJ, *et al.* Association between children's experience of socioeconomic disadvantage and adult health: a life-course study. *Lancet* 2002;**360**:1640–5. doi:10.1016/S0140-6736(02)11602-3

27 Thomson WM, Mackay TD. Child dental caries patterns described using a combination of area-based and household-based socio-economic status measures. *Community Dental Health* 2004;**21**:285–90.

28 Sagheri D, McLoughlin J, Clarkson JJ. The prevalence of dental caries and fissure sealants in 12 year old children by disadvantaged status in Dublin (Ireland). *Community Dental Health* 2009;**26**:32–7.

29 Van den Branden S, Van den Broucke S, Leroy R, *et al.* Effects of time and socio-economic status on the determinants of oral health-related behaviours of parents of preschool children. *European Journal of Oral Sciences* 2012;**120**:153–60.

30 Jung SH, Watt RG, Sheiham A, *et al.* Exploring pathways for socioeconomic inequalities in self-reported oral symptoms among Korean adolescents. *Community Dentistry and Oral Epidemiology* 2011;**39**:221–9.

31 Ravaghi V, Underwood M, Marinho V, *et al.* Socioeconomic status and self-reported oral health in Iranian adolescents: The role of selected oral health behaviors and psychological factors. *Journal of Public Health Dentistry* Published Online First: 2012.

32 Sanders AE, Spencer a J, Slade GD. Evaluating the role of dental behaviour in oral health inequalities. *Community dentistry and oral epidemiology* 2006;**34**:71–9. doi:10.1111/j.1600-0528.2006.00261.x

33 Vyas S, Kumaranayake L. Constructing socio-economic status indices: how to use principal components analysis. *Health policy and planning* 2006;**21**:459–68. doi:10.1093/heapol/czl029

34 Berry HL, Welsh JA. Social capital and health in Australia: An overview from the household, income and labour dynamics in Australia survey. *Social science & medicine (1982)* 2010;**70**:588–96. doi:10.1016/j.socscimed.2009.10.012

35 Putnam R. *Bowling Alone: The Collapse and Revival of American Community*. New York: : Simon and Schuster 2000.

36 Kahn RL, Antonucci TC. Convoys over the lifecourse; attachment roles and social support. In: Baltes PB, Brim O, eds. *Life span development and behaviour*. New York: : Academic Press 1980. 253–86.

37 WHO. The Ottawa Charter for Health Promotion. Geneva: 1986.

For peer review only - http://bmjopen.bmj.com/site/about/guidelines.xhtml

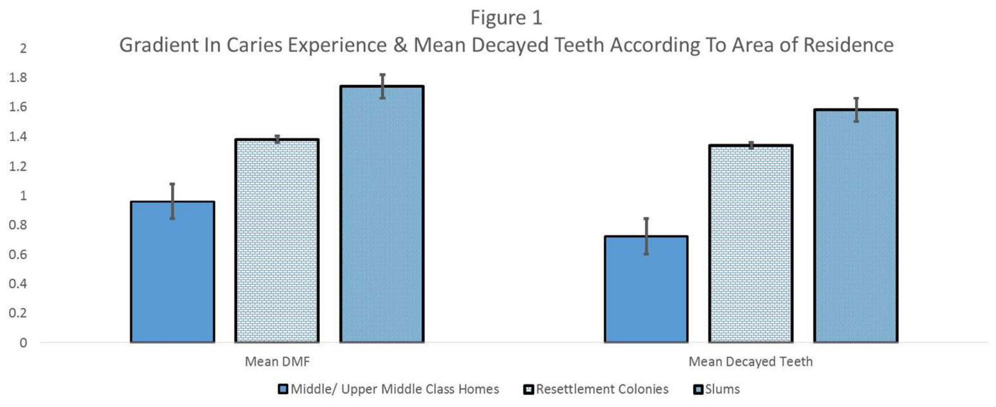

Gradient In Caries Experience & Mean Decayed Teeth According To Area of Residence

146x60mm (300 x 300 DPI)

For peer review only

STROBE Statement—checklist of items that should be included in reports of observational studies

	Item No	Recommendation	Included in Text
**Title and abstract**	1	(*a*) Indicate the study's design with a commonly used term in the title or the abstract	Yes
		(*b*) Provide in the abstract an informative and balanced summary of what was done and what was found	Yes
**Introduction**			
Background/rationale	2	Explain the scientific background and rationale for the investigation being reported	Yes
Objectives	3	State specific objectives, including any prespecified hypotheses	Yes
**Methods**			
Study design	4	Present key elements of study design early in the paper	Yes
Setting	5	Describe the setting, locations, and relevant dates, including periods of recruitment, exposure, follow-up, and data collection	Yes
Participants	6	(*a*) *Cohort study*—Give the eligibility criteria, and the sources and methods of selection of participants. Describe methods of follow-up *Case-control study*—Give the eligibility criteria, and the sources and methods of case ascertainment and control selection. Give the rationale for the choice of cases and controls *Cross-sectional study*—Give the eligibility criteria, and the sources and methods of selection of participants	Yes
		(*b*) *Cohort study*—For matched studies, give matching criteria and number of exposed and unexposed *Case-control study*—For matched studies, give matching criteria and the number of controls per case	NA
Variables	7	Clearly define all outcomes, exposures, predictors, potential confounders, and effect modifiers. Give diagnostic criteria, if applicable	Yes
Data sources/ measurement	8*	For each variable of interest, give sources of data and details of methods of assessment (measurement). Describe comparability of assessment methods if there is more than one group	*Yes*
Bias	9	Describe any efforts to address potential sources of bias	Yes
Study size	10	Explain how the study size was arrived at	Yes

For peer review only

Quantitative variables	11	Explain how quantitative variables were handled in the analyses. If applicable, describe which groupings were chosen and why	Yes
Statistical methods	12	(*a*) Describe all statistical methods, including those used to control for confounding	Yes
		(*b*) Describe any methods used to examine subgroups and interactions	NA
		(*c*) Explain how missing data were addressed	NA
		(*d*) *Cohort study*—If applicable, explain how loss to follow-up was addressed<br>*Case-control study*—If applicable, explain how matching of cases and controls was addressed<br>*Cross-sectional study*—If applicable, describe analytical methods taking account of sampling strategy	Yes
		(*e*) Describe any sensitivity analyses	

Continued on next page

For peer review only

**Results**			
Participants	13*	(a) Report numbers of individuals at each stage of study—eg numbers potentially eligible, examined for eligibility, confirmed eligible, included in the study, completing follow-up, and analysed	Yes
		(b) Give reasons for non-participation at each stage	NA
		(c) Consider use of a flow diagram	NA
Descriptive data	14*	(a) Give characteristics of study participants (eg demographic, clinical, social) and information on exposures and potential confounders	Yes
		(b) Indicate number of participants with missing data for each variable of interest	
		(c) *Cohort study*—Summarise follow-up time (eg, average and total amount)	
Outcome data	15*	*Cohort study*—Report numbers of outcome events or summary measures over time	
		Case-control study—Report numbers in each exposure category, or summary measures of exposure	
		Cross-sectional study—Report numbers of outcome events or summary measures	*Yes*
Main results	16	(*a*) Give unadjusted estimates and, if applicable, confounder-adjusted estimates and their precision (eg, 95% confidence interval). Make clear which confounders were adjusted for and why they were included	Yes
		(*b*) Report category boundaries when continuous variables were categorized	
		(*c*) If relevant, consider translating estimates of relative risk into absolute risk for a meaningful time period	
Other analyses	17	Report other analyses done—eg analyses of subgroups and interactions, and sensitivity analyses	Yes
**Discussion**			
Key results	18	Summarise key results with reference to study objectives	Yes
Limitations	19	Discuss limitations of the study, taking into account sources of potential bias or imprecision. Discuss both direction and magnitude of any potential bias	Yes
Interpretation	20	Give a cautious overall interpretation of	Yes

For peer review only

		results considering objectives, limitations, multiplicity of analyses, results from similar studies, and other relevant evidence	
Generalisability	21	Discuss the generalisability (external validity) of the study results	Yes
**Other information**			
Funding	22	Give the source of funding and the role of the funders for the present study and, if applicable, for the original study on which the present article is based	Yes

*Give information separately for cases and controls in case-control studies and, if applicable, for exposed and unexposed groups in cohort and cross-sectional studies.

Note: An Explanation and Elaboration article discusses each checklist item and gives methodological background and published examples of transparent reporting. The STROBE checklist is best used in conjunction with this article (freely available on the Web sites of PLoS Medicine at http://www.plosmedicine.org/, Annals of Internal Medicine at http://www.annals.org/, and Epidemiology at http://www.epidem.com/). Information on the STROBE Initiative is available at www.strobe-statement.org.

For peer review only
